# Health and Environmental Impacts of Cyanobacteria and Cyanotoxins from Freshwater to Seawater

**DOI:** 10.3390/toxins17030126

**Published:** 2025-03-07

**Authors:** Tamara Villalobos, Benjamín Suárez-Isla, Carlos Garcia

**Affiliations:** Laboratory of Marine Toxins, Physiology and Biophysics Programme, Faculty of Medicine, University of Chile, Santiago 8330111, Chile; tamara.villalobos@uchile.cl (T.V.); bsuarez@u.uchile.cl (B.S.-I.)

**Keywords:** cyanobacteria, cyanotoxins, bioaccumulation, health risk, human, ecosystem, microcystin, anatoxins, cylindrospermopsins, nodularins, stakeholders

## Abstract

Cyanobacterial harmful algal blooms (cyanoHABs) are a natural phenomenon produced mainly by the interaction between natural and anthropogenic events. CyanoHABs are characterized by the production of cyanotoxins that can have harmful effects on different species within the food web and even affect human health. Among the most prevalent toxin groups worldwide are microcystins (MCs), anatoxins (ATXs), cylindrospermopsins (CYNs) and nodularins (NODs), which are characterized as toxins with hepatotoxic, neurotoxic, and cytotoxic effects. This review summarizes and analyzes research on the influence of cyanoHABs, the main toxin-producing cyanobacteria and the most prevalent cyanotoxins in freshwater and marine bodies, highlighting their global occurrence, toxicology, and bioaccumulation dynamics in vectors of the food web, and the main cases of acute and chronic intoxications in humans. This review is useful for understanding the dynamics of cyanoHABs’ interaction with the ecosystem and their impact on human health, and how the implementation of a surveillance and management framework for cyanobacteria and cyanotoxins could generate vital information for stakeholders to establish health guidelines on the risks and hazards of cyanoHABs for the ecosystem and humans.

## 1. Introduction

Cyanobacteria are phytoplanktonic, prokaryotic, and photoautotrophic (Gram-negative) microorganisms, and have been producers of atmospheric oxygen on Earth for about 3.5 billion years, a period in which they have adapted to different climatic, geochemical, and anthropogenic changes [1,2,3]. These blue-green algae represent the primary producers in aquatic ecosystems, being considered natural inhabitants of rocks, soils, and diverse environments such as freshwater, estuaries, and marine waters [4]. Their intense proliferative capacities tend to produce biomass, which causes turbidity, discoloration of the water, and, sometimes, foam formation, processes known as blooms [5,6,7,8,9,10,11].

Blooms, specifically related to cyanobacteria, are defined as “the increase of biomass in a waterbody (chlorophyll-α concentration) over a relatively short period of time (between a few days and 1 to 2 weeks) and characterized by the predominance (>80%) of a single species or a few species within the phytoplankton community” [12,13,14]. Through this process, cyanobacteria can reach concentrations of more than 10^6^ cells per liter of water [15].

The last century has been marked by a constant and excessive increase in the levels of greenhouse gases and nutrient inputs. These pollutants end up in freshwater courses such as lakes and rivers, resulting in eutrophication processes, defined from a natural point of view as an excessive increase in primary production derived from a high rate of photosynthesis, which is triggered by the natural discharge of nitrogen and phosphorus, which are carried away by rain and surface waters that erode Earth’s surface [16,17].

**Figure 1:**
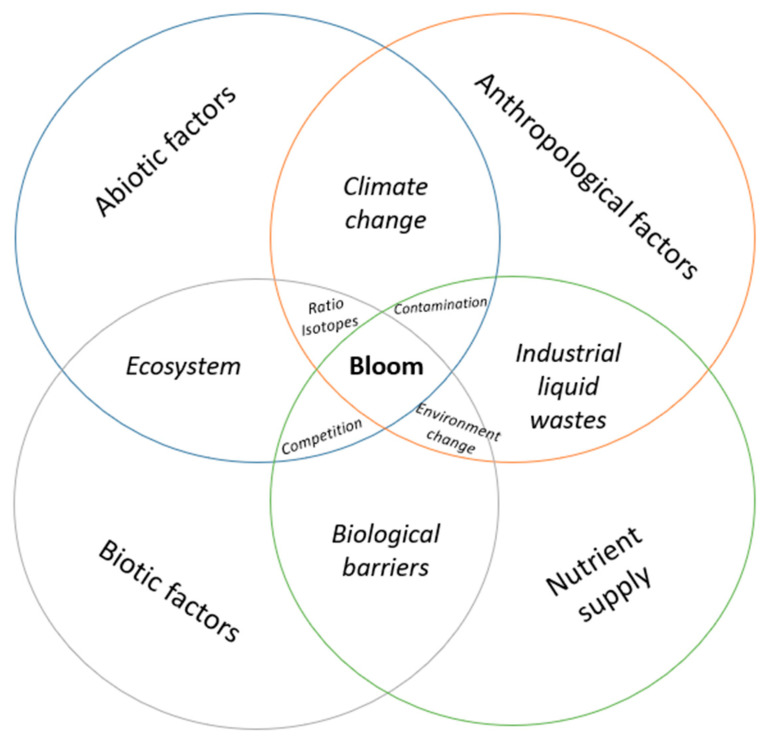
Diagram of factors influencing incidence of cyanoHABs.

In addition, anthropogenic activities associated with high agricultural activity, high levels of industrial waste, and the demographic growth of cities have caused gradual alterations in the physical–chemical state of lentic and lotic water bodies, causing the alteration of their trophic states (Figure 1) [3], generating the decomposition of organic matter and a decrease in the concentration of dissolved oxygen in the water, and consequently causing the death of different species associated with the aquatic ecosystem, such as benthic invertebrates and fish [6,18,19].

**Figure 2:**
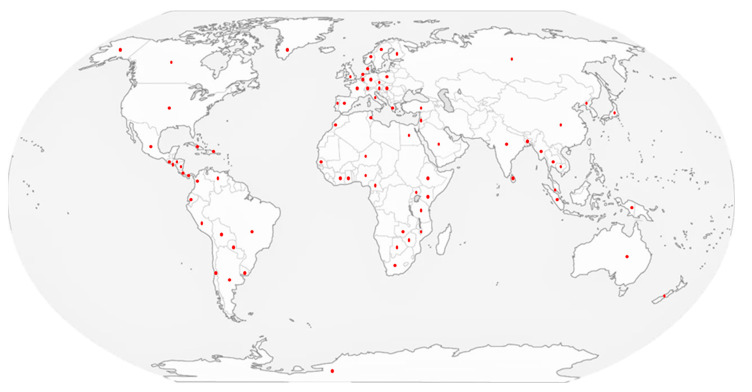
Map of global distribution of cyanoHABs (denoted by red dots).

Blooms tend to occur seasonally, usually in spring and summer, and are the result of excessive nutrient enrichment, particularly nitrogen and phosphorus [19,20]. This, together with favorable conditions of temperature, light penetration in the water column, water pH, conductivity (salinity), water turnover time in lotic zones, food web variability, and carbon dioxide availability, favors the displacement of the phytoplankton community towards cyanobacteria, which can form dense and recurrent blooms [4,5,6,18,19,20,21,22]. The sum of these factors plus climatic variability have allowed certain taxa of freshwater cyanobacteria to become more frequent at different latitudes and/or in specific sectors or regions of most countries, and their incidence has tended to increase due to global changes generated by anthropogenic activities (Figure 2) [1,12,23,24,25,26,27,28,29,30].

The greatest danger associated with cyanobacteria is that under certain conditions, cyanobacteria produce bioactive metabolites called cyanotoxins. Because cyanotoxins pose risks to aquatic organisms and humans, blooms that produce these chemicals are called “cyanobacterial harmful algal blooms” (cyanoHABs) [12,14,31,32].

About 3000 species of cyanobacteria are currently known; however, not all of them produce toxins [19,33], since the dynamics between physicochemical parameters, genetic factors between species, and the physiological state of cells can affect/alter cyanotoxin synthesis (cell quota), thus allowing an interaction between toxic and non-toxic genotypes to occur environmentally [34,35,36]. However, paleolimnological analyses allow us to determine that both the abundance and toxicity of cyanoHABs date back millennia [37,38,39,40,41].

From an environmental point of view and according to biotic and abiotic interaction conditions, it is possible that cyanobacteria alternate their life cycle between the benthos and the pelagic zones, allowing inoculation to promote flowering or to settle in the sediment (seed bank) until the right conditions for growth are in place [42,43,44,45,46,47,48]. This process is key to cyanotoxin content, as cyanotoxins are not passively excreted by cyanobacteria and are usually released into the aquatic environment during cell death and lysis. However, some species of cyanobacteria can release toxins into the water without rupturing or dying (e.g., *Cylindrospermopsis* sp.) [3,49].

The global impacts of cyanoHABs and their toxins generate high socioeconomic and ecological costs, affecting without limitation drinking water sources, lake and marine resources, agriculture, transportation, tourism, trade, the food web, and ecosystems [50,51]. For these reasons, the World Health Organization (WHO) has developed guidelines based on the densities and toxicities of cyanoHABs in water, establishing that different types of exposures can pose a significant risk to human health [33,52,53].

Although there is important scientific information on the subject, there are still questions regarding the following: How are toxins assimilated at different trophic levels? Are they events that only affect inland water bodies? Is there any relationship between cyanoHABs and marine vectors? How relevant is the impact of cyanoHAB exposure on human intoxication? How can decision makers be guided to understand the risks and hazards of cyanoHABs to the environment and public health?

The main objectives of this paper are to examine and establish the dynamics of cyanoHABs and cyanotoxins in freshwater and estuarine bodies of water, including the trophic network, in order to propose actions that will allow the application of quality control and management methods in inland water bodies.

For this review, we compiled data from published scientific studies investigating the occurrence of the most prevalent cyanobacteria/cyanotoxins worldwide, their toxicology, the assimilation and interaction between trophic networks in freshwater and marine species, the main cases of intoxication in humans, and the risks and hazards associated with cyanoHABs and cyanotoxins. A wide range of environmental, toxicological, and other scientific literature was explored via the Scopus database, which includes PubMed, Web of Science, and ScienceDirect.

## 2. Cyanotoxins

Cyanotoxins correspond to secondary metabolites, i.e., they are intermediates or metabolic products that do not play a vital role in the growth, development, and/or reproduction of cyanobacteria; therefore, it is suggested that they have an auxiliary purpose of responding to environmental stress, or that they are synthesized to act as a defense mechanism against the threat of other microalgae species competing for light, nutrients, and habitats [12,54,55,56].

The detection of cyanotoxin concentrations in processes associated with cyanoHABs depends primarily on four factors: (1) species abundance; (2) the abundance of genotypes (toxigenic); (3) the type(s) of toxin(s) that can be produced by the taxon; and (4) the cellular quota of toxins [57,58,59]. Thus, cyanotoxins that are preferentially found intracellularly can be detected in the water, which will be favored according to the chemical nature of the toxins and the stage of bloom development, this process being more characteristic in stages of the senescence or decline of blooms [59,60,61,62,63].

This release of cyanotoxins into the environment can have significant effects on aquatic species, including decreased survival rates, altered development, larval mortality, reduced feeding, and death [12,64]. Harmful effects may be enhanced, since some cyanobacterial taxa can produce a wide variety of cyanotoxins and may even produce different groups of toxins [12,65]. These variables favor the possibility that cyanotoxins can bioaccumulate (process by which toxins enter the food web by building up in individual organisms) in different aquatic vectors, favoring their transfer through the trophic network until they reach people, which can cause different clinical pictures of acute or severe intoxication [12,36,66,67,68].

### 2.1. Hepatotoxins

#### 2.1.1. Microcystins (MCs)

**Figure 3:**
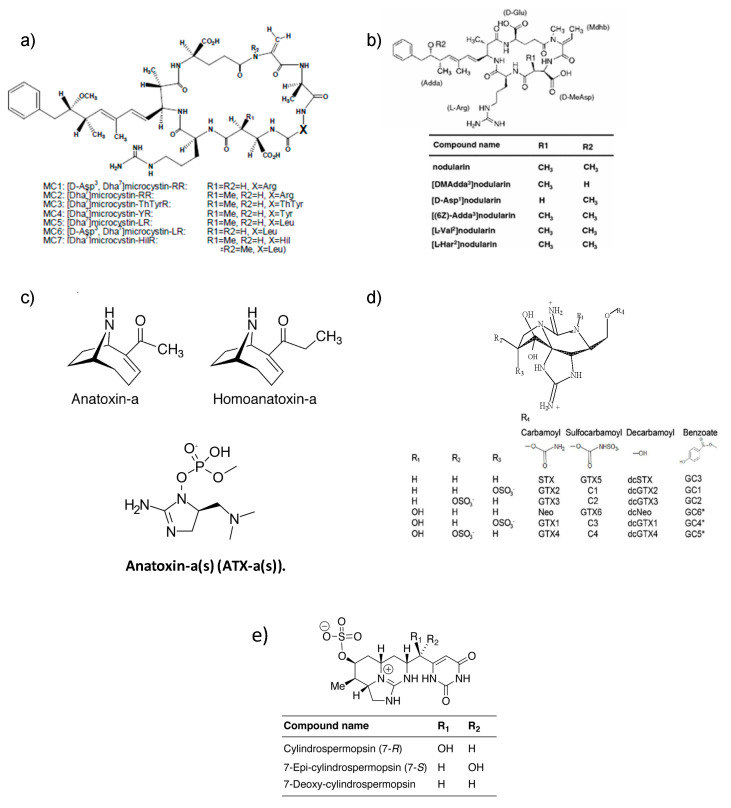
Structure of cyanotoxin groups. (**a**) Microcystins (MCs); (**b**) nodularins (NODs); (**c**) anatoxins (ATXs); (**d**) saxitoxins (STXs); (**e**) cylindrospermopsins (CYNs).

Microcystins correspond to a group of cyclic heptapeptides which have seven amino acids (ciclo-(D-alaninel-X2-DMeAsp3-Z4-Adda5-D-glutamato6-Mdha7)) (M.W. 881–1360 Da) (Figure 3a) [69]. Methylations, hydroxylations, epimerizations, and amino acid substitutions give rise to structural diversity. So far, more than 300 different analogs have been identified in lakes or cell cultures, the most prevalent analogs being MC-LR, MC-RR, and MC-YR, with MC-LR standing out for its high toxicity (Figure 3a) [70,71,72,73,74,75].

MCs are stable, non-volatile, water-soluble molecules produced as secondary metabolites by different genera of cyanobacteria at levels of up to 1% by mass (Table 1) [67,70,76].

This group of cyanotoxins is characterized by having different types of toxic levels according to the analog detected (LD_50_ MC-LR y-RR 50 μg/kg and 600 μg/kg b.w., respectively) [76,77,78]. *Microcystis aeruginosa* is the dominant producer of MCs; however, it has also been detected in taxa such as *Anabaena* sp., *Oscillatoria* sp., and *Planktothrix* sp. (Table 2) [79,80].

At the cellular level, MCs cause specific damage to liver cells by inhibiting protein phosphatases (PP1 and PP2A), resulting in the hyperphosphorylation of cellular proteins [81]. This occurs because the amino acid ADDA is covalently bound to the cysteine residue that both enzymes have in their catalytic center, Cys-266 in the case of PP1 [82,83,84] and Cys-273 in the case of PP2A [85]. Thus, it is the amino acid ADDA that defines the toxicity of MCs [71,86,87,88,89].

**Table 1:** Characteristics of freshwater cyanobacteria and cyanotoxins and their toxic effects.

Cyanotoxin	Cyanobacterial-Producers	Cell Quota	Toxic Effects	Cyanotoxin-Producers	References
Microcystins (MCs)	*Aphanizomenon* *Dolichospermum* *Limnothrix* *Microcystis* *Nostoc* *Oscillatoria* *Phormidium* *Planktothrix* *Gloeotrichia* *Hapalosiphon* *Radiocystis*	<150 to 850 fg/cell	diarrhea, vomiting, stomach cramps, nausea, acute liver failure, chronic kidney disease, respiratory symptoms, abdominal pain, sore throat, dry cough, blistering at the mouth, headache, flulike symptoms, irritation and rashes (colorectal cancer). ^1^	*Microcystis* spp., *M. aeruginosa*, *M. viridis*, *Dolichospermum* sp., *Anabaena flos-aquae*, *A. subcylindrica*, *A. variabilis*, *Oscillatoria (Planktothrix) agardhii*, *Nostoc* sp., *Nostoc spongiaeforme*, *Anabaenopsis* sp., *Hapalosiphon* sp., *Gloeotrichia echinulata*, *Plectonema boryanum*, *Phormidium corium*, *Phormidium splendidum*, *Rivularia biasolettiana*, *R. haematites*, *Tolypothrix distorta*, *Arthrospira fusiformis*.	[12,90,91,92]
Nodularins (NODs) ^2^	*Nodularia Noctoc*	60–500 fg/cel	Allergic reactions, skin rashes, gastrointestinal illness, nausea, liver damage, bleeding.	*Nodularia spumigena*, *Nostoc* sp.	[2,12,91]
Cylindrospermopsins (CYNs)	*Anabaena* *Cylindrospermopsis* *Aphanizomenon* *Chrysosporum* *Raphidiopsis* *Umezakia*	<1.9 to 196 fg/cell	Nausea, vomiting, diarrhea, stomach cramps, hepatomegaly, kidney dysfunction.	*Cylindrospermopsis raciborskii*, *Aphanizomenon ovalisporum*, *Dolichospermum* sp., *Anabaena lapponica*, *Raphidiopsis curvata*, *Umezakia natans*.	[12,90,91]
Anatoxin-a (ATX)	*Anabaena* *Aphanizomenon* *Cylindrospermum* *Microcystis* *Oscillatoria* *Phormidium* *Planktothrix*	0.1–500 fg/cell	Convulsions, fatigue, paralysis, respiratory failure.	*Arthrospira fusiformis*, *Anabaena* spp., *Aphanizomenon* sp., *Phormidium* sp., *Anabaena flos-aquae*, *Anabaena planktonica*, *Cylindrospermum* sp., *Oscillatoria* sp., *Raphidiopsis meditteranea*, *Phormidium formosum*.	[90,91]
Saxitoxins (STXs)	*Cuspidothrix* *Dolichospermum* *Microseira* *RaphidiopsisPlanktothrix Oxynema*	120–1300 fg/cell	Tingling sensation around the lips, convulsions, headaches, dizziness, nausea, vomiting fatigue, paralysis, respiratory failure.	*Aphanizomenon flos-aquae*, *Microseira* sp. *Dolichospermum circinale*, *Cylindrospermopsis raciborskii*, *Planktothrix* sp.	[90,91]

^1^ Several human epidemiological studies from China have reported an association between liver or colon cancer and the consumption of drinking water from surface waters containing cyanobacteria and microcystins. In these studies, individuals with liver cancer were also exposed to aflatoxins and hepatitis, both risk factors for liver cancer. The results from this work demonstrate a possible association, but do not directly determine a link between microcystin exposure and liver cancer. ^2^ No epidemiological studies have explicitly investigated the relationship between NOD exposure and health outcomes at the population level.

**Table 2:** Toxicity of cyanotoxins.

Cyanotoxin	Primary Toxicity	Mode of Action	Lifetime Drinking Water (µg/L)	Short-Term Drinking Water (µg/L)	Recreational Water (µg/L)	LD_50_	TDI (µg/kg/day)	NOAEL (µg/kg bw/day)	LOAEL (µg/kg bw/day)	Classification	References
Microcystins	Hepatotoxicity	Inhibition of protein phosphatases	1.0	12.0	24.0	5.0–10.9 (oral; µg/kg)	0.04	40.0	50.0	Group 2B—possibly carcinogenic to humans.	[2,53,87,93,94,95]
Nodularins	Hepatotoxicity	Inhibition of protein phosphatases	1.0	12.0 ^1^	n.d.	50.0 (intraperitoneal; µg/kg)	0.04	100.0	n.d.	Group 3— not classifiable as to its carcinogenicity to humans.	[50,53,93,96]
Cylindrospermopsin	Hepatotoxicity	Inhibition of protein phosphatases	0.7 (0.01)	3.0	6.0	4.4–6.9 (oral; µg/kg)	0.03 ^2^	30.0	150.0	n.d. (potential for carcinogenicity)	[52,53,97,98]
Anatoxin-a	Neurotoxicity	Nicotinic acetylcholine receptor agonists	1.0	30 ^3^	60.0	>5000.0 (oral; µg/kg)	0.1 ^2^	98.0	n.d.	n.d.	[52,53,97,99]
Anatoxin-a(s)	Neurotoxicity	Inhibition of acetylcholinesterase	1.0	n.d.	n.d.	20.0–40.0 (intraperitoneal; µg/kg)	n.d	n.d	n.d	n.d.	[52,100,101,102]
Saxitoxins	Neurotoxicity	Blocking of sodium channels	3.0	0.3	30.0	35.0 (oral; µg/kg)	0.05 ^2^	0.5	1.5	n.d.	[52,53,97,101,103]

^1^ Sufficient toxicological data are lacking for the derivation of a guideline value (GV) for nodularins. Tentatively, the microcystin GV can be applied due to the structural and toxicological similarity between microcystins and nodularins. ^2^ Oregon Health Authority (OHA). ^3^ Valore referential short-term and lifetime exposure GVs were not developed, and short-term exceedances of the acute GV should not be permitted. n.d.: not detected.

Exposure to these toxins has caused significant effects and deaths at the trophic level, including small planktonic invertebrates, fish, other fish species [104,105], and vertebrates (dogs, cows, sheep, otters, and horses) [1,106,107,108]. The main route of toxic action is through ingestion, although it has been proposed that inhalation may be another route of absorption of MCs [109,110,111].

The major source of exposure to MCs for humans is from drinking water, although other pathways, such as food, interaction with recreational waters, and nutritional supplements, may be significant [76,90]. These cyanotoxins, when ingested, are transported throughout the body, where they enter the cells through organic anion transport polypeptides (OATP) present in the membranes. The main target of action is the liver [112]; however, MCs can also affect other tissues such as the kidneys, colon, brain, lungs, and heart [2,50,113,114]. In addition, MC-LR modulates the expression of tumor necrosis factor α (TNF-α) and proto-oncogenes such as *c-jun*, *jun B*, *jun D*, *c-fos*, and *c-myc* [2,14].

The World Health Organization (WHO) has established a tolerable daily intake (TDI) in drinking water of 1.0 μg/L for MC-LR, while for recreational activities, a level of 20 μg/L MC-LR (value according to an intake volume of 0.2 L per day) has been set (Table 3) [90]. These levels have been accepted in most countries, with MC-LR being classified as “possibly carcinogenic to humans” in 2010 [57,101,115].

#### 2.1.2. Nodularins (NODs)

This group of toxins corresponds to cyclic peptides consisting of five amino acids, including the characteristic amino acid ADDA, which assigns the structural feature to NOD toxins [116]. Structural variations have given rise to at least 10 variants of NODs being described (Figure 3b) [69,117]. Nodularin is the most common pentapeptide (M.W. 825 Da) of general structure cyclo-(D-MeAspl-L-arginine2-Adda3-D glutamate4-Mdhb5), and Mdhb is 2-(methylamino)-2-dehydrobutyric acid [34,118,119]. The most common analog corresponds to NOD-R, cycle (-D-erythro-b-methylAsp (iso)-L-Arg-Adda-D-Glu (iso)-2-(methylamino)-2-(Z)-dehydrobutyric acid), where ADDA stands for 3-amino-9-methoxy-2,6,8-tri-methyl-10-phenyldeca-4 (E), 6 (E)-dienoic acid [120]. The most characteristic NOD-producing species is *Nodularia spumigena*, which is typical of brackish waters (estuaries), whose blooms have been detected mainly in Europe, South Africa, Canada, Australia, the United States, and New Zealand [50,65,121,122].

NODs share a similar chemical structure to MCs, allowing them to have the same level of toxic action, i.e., inhibit PP1/2A [74,123,124,125,126,127].

Experimental data (i.p. in mice) have established that NODs accumulate mainly in the liver; however, it is possible to detect significant levels in the blood and intestines, where they enter via diffusion through non-specific organic anion transporters, using the bile acid transport system as a pathway [65,91]. Their toxicity at the hepatic level is characterized by causing disorganization of the cytoskeleton, lipid peroxidation, loss of membrane integrity, cellular vesicle formation, cell disruption, necrosis, intrahepatic hemorrhage, and apoptosis, all of which provide the ideal scenario for death by hemorrhagic shock. Additionally, NODs induce oxidative stress and produce reactive oxygen species (ROS), causing damage such as lipid, protein, and DNA peroxidation [65,126].

Although several studies have shown that NODs induce the expression of TNF-α and proto-oncogenes, this group of cyanotoxins has been classified by IARC as “animal carcinogenic but not classifiable as to its carcinogenicity to humans” [3,69].

NOD-associated blooms have caused extensive hemorrhaging and deaths in animals, but to date, there are no epidemiological data that can be associated with human intoxication [12]. However, different countries have established health regulations that set maximum limits for both cyanobacteria in recreational waters and levels of cyanotoxins in water intended for human consumption (Table 2) [3,57,86].

### 2.2. Neurotoxins

#### 2.2.1. Anatoxins (ATXs)

Anatoxin-a (ATX, M.W. 165 Da) and homo-anatoxin-a (HTX, M.W. 179 Da) represent the most characteristic bicyclic alkaloids of this group (Figure 3c) [97]. These toxins are produced by cyanobacteria of different genera (Table 1) [12,128,129,130]. The prevalence of this group of cyanotoxins is low; however, ATX has been detected in blooms at levels of ≈154 μg/L, but like other toxic groups, the release of cyanotoxins to the medium occurs only at senescence stages of the bloom [76,131].

Their chemical characteristics confer high solubility and stability in darkness, and they possess a half-life of ≈1–2 h in the presence of high light intensity [99] and ≈14 days under normal light conditions at basic pH [60,132]. However, once released into an aqueous medium, these cyanotoxins can be weakly adsorbed on sandy sediments and more strongly on sediments rich in clay and organic matter, the latter being characterized as promoters of toxin sorption (ATX-a) [56,133].

ATXs bind irreversibly to acetylcholine receptors, causing depolarization of postsynaptic neuronal cells [102,134]. Symptoms produced by ATX (LD_50_ 260 μg/kg b.w.) include convulsions, limb spasms, and eventual paralysis, leading to death in ≈7 min (Table 1) [74,101,135].

One of the most widely described analogs currently corresponds to anatoxin-a(s) (ATX_(s)_), a neurotoxic alkaloid (M.W. 252 Da), whose chemical characteristic is that it is an N-hydroxyguanidine methylphosphate ester that causes the irreversible inhibition of acetylcholinesterase [12,136], causing symptoms in animals such as convulsions, urinary incontinence, respiratory distress, and hypersalivation, this being the characteristic symptom that assigns the term “s” to the analog [56,137,138,139,140,141].

To date, no epidemiological data have been recorded associating human poisoning with this group of cyanotoxins [76], but it has been detected in water bodies in the U.S.A. and Canada, where toxicosis has been observed in dogs, pigs, and poultry, with time to death ranging from 5 to 30 min [50].

#### 2.2.2. Saxitoxins (STXs)

The saxitoxin group (STX-group) corresponds to polar chemical compounds constituted by a unit called imidazoline, which, according to the modification of some of its functional groups, allows them to be divided into three characteristic groups: carbamoyl-toxins, N-sulfocarbamoyl-toxins, and descarbamoyl-toxins. These neurotoxins possess high affinity for voltage-dependent sodium channels, causing muscle paralysis by blocking the nerve impulse [103,142,143]. (Figure 3d).

STX-group toxins are characterized by being mainly associated with harmful algal bloom events (HABs) in the sea (dinoflagellates) [144] and also by having a high prevalence in marine hydrobiological resources, specifically gonyautoxin analogs (GTX; GTX3/GTX2 and GTX4/GTX1), which tend to predominate in the toxin profile in shellfish (75–85%) [144,145,146]. However, these toxins have also been identified in different cyanobacterial genera (*Cylindrospermopsis*, *Dolichospermum*, *Aphanizomenon*, *Planktothrix*, and *Lyngbya*) (Table 1) [74,147,148].

The half-life of saxitoxin (STX) depends on pH and temperature; they are stable at 25 °C and neutral pH, and can be detected in water intended for irrigation and in rivers in the range of ~9 to 28 days post-bloom [56,149]. They exhibit high chemical stability at acidic pH (~2–4), and high degradation at extremely alkaline pH [150,151,152,153].

These toxins have been detected and associated with intense blooms, reaching levels of 193 μg/L in water and with toxicities in different vectors of approximately 4466 μg STX equiv/g dry weight [12,101,154,155,156,157], conducive to the ideal scenario for human exposure through vector consumption and/or recreational activities.

Toxic events associated with intoxication in humans by STX-group toxins have demonstrated a high rate of biotransformation of the analogs [144,156,157,158,159,160]. The identification of STX analogs is regulated by international regulations only in products for consumption of marine origin, but not in freshwater vectors [161]. However, some countries (Australia, Brazil, and New Zealand) have established a maximum permissible level of 3.0 μg/L for drinking water (Table 3) [101,162,163].

### 2.3. Cytotoxin

#### Cylindrospermopsins (CYNs)

This group of cyanotoxins is characterized as tricyclic alkaloids consisting of a tricyclic guanidine group combined with a hydroxyethyl uracil [98]. Cylindrospermopsin (CYN, M.W. 415.43 Da) is the most prevalent analog detected and has been classified as a cytotoxin, since it can affect both the liver (hepatotoxic) and the nervous system (neurotoxic) [2,76]. Modifications in specific groups produce toxic variants such as 7-epi-CYN, 7-deoxy-CYN, 7-deoxy-sulfo-CYN, and 7-deoxy-sulfo-12-acetyl-CYN (Figure 3d) [69,164,165,166].

*Cylindrospermopsin raciborskii* corresponds to the most studied species of this group, and is characterized by being associated with subtropical and tropical habitats; however, it has been classified as an invasive species due to its identification in other latitudes worldwide [74]. Additionally, this species has been associated with STX-group production [3,167].

This group of analogs, as well as other cyanotoxins, are characterized by being water-soluble, highlighting that the areas associated with blooms are characterized by high levels of extracellular toxins, detected in an average range of 20–95% of net production [61,168,169,170,171,172].

The main target organ is the liver, although toxic effects have been described in kidneys, lungs, heart, and thymus [74,173,174,175,176]. The molecular structure of CYN confers hydrophilic characteristics; therefore, its intestinal absorption and incorporation into hepatocytes is mediated by active transport systems, such as the bile acid transport system [12,177,178].

In vitro studies have demonstrated the mutagenicity of CYN [179,180,181]. However, no information is available on the carcinogenicity of this group in humans, nor has tumor-initiating activity been established [166,182,183,184].

Environmentally, toxic levels of CYN have been reported in surface waters at concentrations of ~173 μg/L [12] and sometimes with the coexistence of MCs [70]. These high environmental levels have suggested that this group of cyanotoxins possess the capacity to disrupt the antioxidant system and/or cause oxidative stress in a wide variety of aquatic animals [36].

To date, only New Zealand and Cuba have developed monitoring guidelines for risks associated with benthic toxic blooms, even though toxin concentrations are not detailed, given the constant spatial and temporal variation in blooms [185,186,187].

**Table 3:** Drinking and recreational water guidelines of different cyanotoxins worldwide.

Country/Organization	Cyanotoxin	Maximum Concentration in Drinking Water	Maximum Concentration in Recreational Water	References
WHO	Microcystin-LR	1.0 μg/L	24.0 μg/L	[188]
	Cylindrospermopsin	0.7 μg/L	6.0 μg/L	
	Saxitoxins	3.0 μg/L	30.0 μg/L	
	Anatoxin-a	3.0 μg/L	60.0 μg/L	
USEPA	Microcystin	0.3 μg/L ^1^ 1.6 μg/L ^2^	<8.0 μg/L	[189,190,191,192]
	Cylindrospermopsin	0.7 μg/L ^1^ 3.0 μg/L ^2^	<15.0 μg/L	
	Saxitoxins	0.3 μg/L ^1^ 1.6 μg/L ^2^	8.0 μg/L	
	Anatoxin-a	0.7 μg/L ^1^ 3.0 μg/L ^2^	15.0 µg/L	
EU	Microcystin-LR	1.0 μg/L		[192]
China	Microcystin-LR	1.0 μg/L ^4^	ND	[190]
Australia	Microcystin	1.3 μg/L	≤10.0 μg/L	[191]
	Nodularin	1.3 μg/L	≤10.0 μg/L	
	Cylindrospermopsin	0.9 μg/L	ND	
	Anatoxin-a	3.1 μg/L	ND	
	Saxitoxins	3.1 μg/L	ND	
Brazil	Microcystin	1.0 μg/L	ND	[193]
	Cylindrospermopsin	15.0 μg/L	ND	
	Saxitoxins	3.0 μg/L (STX equiv.)	ND	
	Nodularin	1.0 μg/L	ND	
Canada	Microcystin-LR	1.5 μg/L ^3^	10 μg/L	[194]
Denmark	Microcystin	1.0 μg/L ^3^	20 μg/L	[195]
Belgium and Luxembourg	Microcystin-LR	1.0 μg/L ^3^	20 μg/L	[196]
France	Microcystin-LR	1.0 μg/L ^3^	≤25.0 μg/L 13.0 μg/L	[197]
Finland	Microcystin	<1.0 µg/L ^3^	ND	[198]
Germany	Microcystin	<1.0 µg/L ^3^	<10.0 µg/L	[199]
Greece	Microcystin-LR	1.0 μg/L ^3^	ND	[200]
Italy	Microcystin	ND ^3^	< 25.0 µg/L	[201]
Poland	Microcystin-LR	1.0 μg/L ^3^	20.0 μg/L	[202]
Czech Republic	Microcystin-LR	1.0 μg/L ^3^	ND	[203]
Portugal	Microcystin-LR	1.0 μg/L ^3^	ND	[204]
Netherlands	Microcystin	1.0 μg/L ^3^	<20.0 µg/L	[205]
New Zealand	Microcystin	1.0 μg/L	≤12.0 μg/L	[206]
	Cylindrospermopsin	1.0 μg/L	ND	
	Saxitoxins	3.0 μg/L	ND	
	Anatoxin-a	6.0 μg/L	ND	
	Anatoxin-a(s)	1.0 μg/L	ND	
	Homoanatoxin-a	2.0 μg/L	ND	
	Nodularin	1.0 μg/L	ND	
Singapore	Microcystin	1.0 μg/L ^4^	ND	[207]
Spain	Microcystin	1.0 μg/L ^4^	ND	[208]
Turkey	Microcystin-LR	1.0 μg/L ^3^	<25.0 µg/L	[209]
	Cylindrospermopsin	1.0 μg/L	ND	
Uruguay	Microcystin-LR	1.0 μg/L	20.0 μg/L	[210]
	Cylindrospermopsin	0.5 μg/L	ND	
	Saxitoxins	3.0 μg/L	ND	
South Africa	Microcystin-LR	ND	<10.0 µg/L	[211]
Perú	Microcystin-LR	1.0 μg/L ^4^	ND	[212]
Argentina	Microcystin-LR	1.0 μg/L ^4^	ND	[213]
Mexico	Microcystin-LR	1.0 μg/L ^4^	ND	[214]
Costa Rica	Microcystin-LR	1.0 μg/L ^4^	ND	[215]
Paraguay	Microcystin-LR	1.0 μg/L ^4^	ND	[216]
Panama	Microcystin-LR	1.0 μg/L ^4^	ND	[217]

ND: not determined. ^1^ Infants and pre-scholar children. ^2^ School-age children and adults. ^3^ European Union guidelines. ^4^ WHO guidelines.

## 3. Food Web Transfer

Given the developmental characteristics of cyanoHABs in aquatic ecosystems, it can be established that they can have varied effects on different aquatic organisms. The first assimilation pathway corresponds to the direct filtration of cyanobacteria by primary consumers (zooplankton, crustaceans, and bivalves), even when the food is considered of poor quality for this trophic segment, as it lacks polyunsaturated fatty acids (PUFA) and essential sterols [138,218,219].

Subsequently, during the cyanoHAB decay phase, the stage of cyanotoxin disposal to the aqueous medium begins, a stage in which all organisms that are part of the food web are directly exposed, in different magnitudes, to the toxins, i.e., from undetected levels to levels of >7000 μg MCs/L; 180 μg CYN/L; 42,300 µg NOD/L; and 1750 μg ATX-a/L [217,220]. This scenario promotes a second pathway, corresponding to the direct filtration of diluted cyanotoxins in the water column. Most toxins are characterized by high hydrophilicity, which apparently favors their distribution in the tissues of the species. However, it is important to note that cyanotoxins do not passively penetrate cell membranes, as they require specific transporters for cellular assimilation [3,70].

### 3.1. Freshwater Vectors

For the first trophic level (primary consumers), which is characterized by the presence of different species of zooplankton and crustaceans, there are data obtained from an environmental point of view associated with cyanoHABs. In fact, species of this trophic level preferentially assimilate senescent cyanobacteria at the bottom of lotic or lentic water bodies, filtering cyanotoxins that may be in different types of sediments. Thus, the types of analogs assimilated can be very diverse according to the chemical stability that toxins can present in the ecosystem. Several studies have established that MCs produce alterations in the development of some zooplankton species. The exposure of MC-LR (10.2 ng/mg b.w.) on *Daphnia* sp. tends to produce alterations in growth rates, as well as to decrease the survival rate of larvae, in addition to the high incidence of deformations in individuals during their development [119,221,222,223].

Likewise, in the case of crustaceans (*Pacifastacus lenisculus*), it has been experimentally established that the species presents high tolerance to exposure to *Microcystis* sp. and that they can also assimilate low levels of MCs without showing toxic effects. However, in species such as *Chasmagnatus granulatus*, gill damage has been observed with concomitant inhibition of Na^+^ K^+^ ATPases and an increase in antioxidant capacity [224].

Regarding CYN exposure, preferential distribution in hepatopancreas and muscle tissues has been experimentally detected in *Cherax quadricarinatus*, with no toxic effects observed [225]. In the case of anatoxin-a, there are few studies on its accumulation in species of this trophic level.

Another important group of affected species is bivalves, benthic invertebrates that represent the most direct connector between cyanoHABs and the food web. These organisms, under normal environmental and physiological conditions, filter an average of 2–3 L/h of water because they are omnivorous organisms, and according to their densities in the water body, they can have an important influence on planktonic species, suspended organic matter, and cyanoHABs [226,227,228]. These freshwater vectors are mainly characterized by ingesting and accumulating cyanotoxins through cell filtration during cyanoHABs. However, certain patterns can modulate the assimilation and filtration process selectively, such as taxon type, cell size, cell morphology, colony formation or a lack thereof, toxicity, and the types of cyanotoxins. Additionally, bivalves may excrete some types of cyanobacteria as pseudofeces, a process that also appears to influence the density of cyanobacteria in the water column (Table 4) [228].

In the case of MCs, the different accumulation rates detected in bivalves are related to the specific assimilation capacities, the developmental stages of each species, and the predominant analog associated with the bloom, which is why the most identified and studied analog is MC-LR [70,101]. However, it has been determined that bivalves show high resistance to the accumulation of MCs and that the degree of accumulation is clearly associated with physiological aspects and vertical distribution in the stratum. Thus, different species in the same body of water can accumulate varying concentrations (0.07–420 μg/g) without showing toxic effects (*Anodonta woodiana*, *Cristaria plicata* and *Unio douglasiae*), while in other vectors (gastropods), exposure may result in the death of the species [231].

Once the toxins are filtered, they are preferentially accumulated in the digestive glands (hepatopancreas), tissue in which the stages of bioconversion and distribution of the analogs are initiated. The bioconversion phase is through GST (*Dreissenia polymorpha* and *Diplodon chilensis*), without consideration of the fact that some analogs are not metabolized, in order to initiate the process of purification of the metabolized analogs (*Diplodon chilensis* > 60%), and also of the non-metabolized fraction [119,299,300,301]. This process predisposes sandy-bottom-habitat species (clams) to leach and accumulate a mixture of free and metabolized toxic analogs, favoring low retention rates and the bioconversion and distribution of cyanotoxins in visceral and non-visceral tissues [119].

The World Health Organization (WHO) has established a tolerable daily intake (TDI) for MCs in seafood of 24 μg/kg wet weight based on a 60 kg person consuming 100 g of seafood per day [302,303].

Regarding CYNs, the levels of these types of cyanotoxins in benthic organisms are very low (*Alathyria pertexta*), with toxicities in the range of 130–560 μg/kg, following the trend of other types of toxins, i.e., with preferential accumulation in the digestive glands with low bioconversion rates [229]. Meanwhile, in other species, variable toxicities have been detected, such as in snails (3.35 ± 1.90 ng/g) and shrimp (0.1–4.3 μg/g) [12,68,251].

The next trophic level comprises different types of fish, which are usually classified as secondary consumers. However, their classification can be refined according to diet; thus, in freshwater bodies, we can find detritivorous, omnivorous, herbivorous, and carnivorous fish, which establishes that the assimilation and transfer of cyanoHABs and their toxins seem to be highly specific according to the predominant type of species in the ecosystem [304]. Several studies have determined that, in the case of MCs, they tend to be transferred into the trophic chain, reaching variable concentrations according to the type of fish feeding. Once ingested, MCs tend to bind covalently to the proteins of different tissues, which allows us to understand the dynamics of the prevalence and interaction of toxins in some tissues. MCs tend to be mostly detected in the liver, intestines, kidneys, and gonads and, to a lesser extent, in muscle (<20 times) [101,248,289,304,305]. Miles et al. [306] establishes that post-exposure MCs have a dual action, being classified as toxic (free) analogs, which are not covalently bound to any protein, and non-toxic analogs, covalently bound to any structure. However, the latter fraction can be released, generating toxic action in a secondary way when circulating in the system. Thus, MCs, according to their molecular mode of action, can generate liver and gill diseases as a consequence of exposure (via PP2A inhibition) [222].

Complementarily, the toxic effects are dependent on the developmental stages of the fish (larvae/juveniles/adults). MCs, in early stages, tend to generate greater alterations in larval development: they decrease growth rates, generate malformations, and produce important alterations in antioxidant capacity, which tends to alter the number of individuals in a specific habitat. In adult individuals, assimilation depends directly on the feeding route (direct vs. vectorial), which, added to the high development of their epithelial layer, their greater metabolic capacity, and their high mobility in the aquatic environment, makes them more resistant [12,307].

Detritivorous, omnivorous, and herbivorous fish feed directly on cyanobacteria and may indirectly ingest cyanotoxins. Thus, after environmental exposure to cyanoHABs, the route of interaction of fish with toxins and cyanoHABs (MCs) is mainly by oral ingestion and secondarily by absorption through the gill epithelium [3,308]. Therefore, the toxic capacity depends largely on the stage of development of the bloom, which will define the type of toxin availability. In the early stages, more intracellular toxins will be available, while in the senescence stage, the toxin availability will be diluted in the water, generating a lower toxic effect. Thus, the route of MC exposure is decisive for its toxic effects in fish, since exposure through the medium would generate a lower effect without causing mortality compared to the oral route [88].

Environmental evaluations of the effect of MCs on different types of fish have determined that omnivorous species such as tilapia (*Oreochromis niloticus*, 0.6–15 μg/kg DW) and silver carp (*Hypophthalamichtys molitrix*, 1.16–17.8 μg/kg DW) have low toxin accumulation rates, since they are characterized by selective feeding, generating the option to exclude themselves from the effects of the bloom of toxic taxa. Complementarily, when comparing omnivorous species (*Perca* sp., 50–130 μg/kg DW) with carnivorous species (*Oncorhynchus* sp., 90.66 ng/g DW), it has been determined that some omnivorous species tend to be more sensitive, which is directly related to their low metabolic capacity (Table 4) [309,310].

Given the incidence of certain species of cyanoHABs in freshwater bodies, concentration data in fish tend to be preferentially oriented towards MCs and to a lesser extent towards CYNs. Thus, data related to CYNs are scarce, but it has been environmentally established that this group of toxins accumulates preferentially in the digestive system and at low concentrations in the muscle. Toxicities also depend on the type of fish and its diet (0.081, 0.42, and 1.2 μg/g in *Bramocharax caballeroi*, *Oreochromis nilaticus*, and *Melanotaenia eachamensis*, respectively) [244,264,311].

Regarding ATX-a, there are few studies on its assimilation and accumulation in fish. However, toxicities have been detected in omnivorous fish (*Cyprinus carpia*) in the range of 0.030–0.768 μg/g [249].

In addition, there is little information regarding the environmental impact of NODs on freshwater organisms, mainly because this type of toxin is preferentially found in estuaries, where the waters are slightly brackish, or in coastal lakes with saline characteristics [65]. However, it has been possible to establish NOD transfer in different fish species in the range of 24–700 μg/kg [119,259,271,312].

Therefore, it can be established that in aquatic ecosystems (lentic and lotic), fish play an important role in the assimilation of cyanoHABs through direct feeding or through the food web [228]. However, although these species are directly and indirectly exposed to the different types of cyanotoxins, the data evidence that their accumulation in tissues in adult individuals is low, a plausible proposal being the high capacity of metabolization and detoxification of most of the ingested cyanotoxins via GTS (MCs, NOD), Cytochrome P450/GST (ATX and ATX_(s)_), and Cytochrome P450 (CYN), thus favoring less toxic and more-water soluble metabolites [138].

However, different levels of cyanotoxin (MCs/CYN) accumulation in biota cannot be predicted based on trophic level, as concentrations depend on several interactions, including the organism’s consumption rate, digestive capacity, and time since exposure. Berry et al. [311], through an evaluation of different types of fish, established a higher concentration of MCs in phytoplanktivorous fish compared to omnivorous and zooplanktivorous fish, suggesting that the accumulation of these toxins varies according to the direct interaction with cyanoHABs and not according to biomagnification processes [311]. Ferrão-Filho et al. [138], through a meta-analysis in species of different trophic levels, confirmed that the ecological data point to a process of biodilution (a decrease in the concentration of a pollutant with a corresponding increase in the trophic level in a body of water) of cyanotoxins (MCs) to the detriment of biomagnification (the process by which toxins are passed from one trophic level to the next within a food web) [138].

Undoubtedly, the high-water solubility of cyanotoxins (MC-LR, CYN and ATX) and the high detoxification capacity in adult individuals are factors that promote the biodilution of toxins in the food web, greatly hindering biomagnification [313]. However, the capacity of some cyanobacterial species to produce more than one type of toxin (MCs/STXs; MCs/CYNs) could affect the metabolic activity of some aquatic species, leading to the biomagnification of some types of specific cyanotoxin analogs, thus constituting a threat to public health [3,138,314]. In addition, it is important to note that a key factor contributing to biodilution is that most primary consumers are omnivores (especially filter feeders), as they consume a mixed diet composed of allochthonous and autochthonous organic matter, which would explain the differences in cyanotoxin bioaccumulation in trophic networks [313].

### 3.2. Marine Vectors

Countries with regulatory norms regarding cyanobacteria and their toxins set sanitary and regular controls in freshwater bodies. However, given the flow dynamics of certain lentic and lotic bodies, it is possible to transfer these microorganisms through currents to the sea, and there are species of cyanobacteria that, under environmental conditions, increase their prevalence in brackish water (*Nodularin* sp.), which allows them to contribute toxins more constantly to the shoreline [41,315]. From this perspective, it is evident that osmotic variation affects the integrity of cyanobacteria, leading to cell lysis with the concomitant release of cyanotoxins into the aqueous medium. Thus, the accumulation of different cyanotoxin analogs in benthic and planktonic marine species is plausible [49,106,316].

In the last 20 years, different investigations have focused on determining the transmission of cyanotoxins from freshwater to marine products, which has allowed the detection of different analogs in bivalve tissues. MCs have been detected in native and cultured mussels, establishing a coherent dispersal dynamic in the freshwater → estuary → seawater transect, which has been corroborated by demonstrating the presence of *Microcystis* sp. taxa with *mcyB* gene expression [69,317,318,319].

The mode of interaction is preferentially through the direct filtration of cyanotoxins, removing the option of the rejection or generation of pseudofeces by marine bivalves, since direct contact with cyanoHABs is very low (>5%). Thus, assimilation, like for other toxins, would follow the same dynamics as in freshwater species, filtration→assimilation→distribution→metabolization→elimination, with the retention and bioconversion capacity of the new toxic analogs generated varying in each species. Thus, different types of cyanotoxins (MCs, CYNs, ATXs, and NODs) have been identified in bivalves from different habitats (rocky stratum and sandy bottom)—such as *Macona blathica* (≥80–320.0 μg/kg), *Mytilus edilus* (0.28–1110 μg/kg), *Mytilus galloprovinciales* (0.7–141.5 ng/kg), and *Mytilus tronssulus* (1.9–32.3 μg/kg) (Table 4) [237,238,241,320].

The accumulation of cyanotoxins could follow a dynamic related to the periods of higher incidence of cyanoHABs, corresponding to spring–summer, which would allow, in some cases and according to the periodicity of blooms, for varied and constant contributions to be made to marine species, allowing filtration to be cumulative in some marine species that stand out for having a high filtration rate (e.g., *Mytilus* sp.). This does not rule out that during the summer period, some species may show complete or partial detoxification/purification processes for some analogs of the different groups of cyanotoxins [236,273,303]. It should be noted that this detoxification/purification process is not definitive and that it is also species-specific, which may mean that in some percentage ranges, there is distribution of toxins to the non-visceral tissues (muscles) of filter feeders. This is the case for CYN, which has been detected in mussels at levels of ~3 mg/kg [317].

Amzil et al. [49], through the EMERGTOX program implemented in France since 2018, showed that it was possible to detect a wide group of unregulated toxins in seafood products (mussels and gastropods), including spirolides, pinnatoxins, gymnodimine, brevetoxins, microcystins, anatoxin, and cylindrospermopsin, among others. In this study, MCs and CYN were detected at concentrations of 9.0 and 18.0 μg/kg, respectively, and ATX was detected for the first time in mussels from the coast of France. In addition, it was possible to determine that the greatest concentrations were found in *Microcystis* sp. The study even identified that the most assimilated toxins corresponded to the dmMC-RR and MC-RR analogs, establishing favorable bioconversion to dmMC-RR in the tissues of plovers [49].

Regarding NODs, these cyanotoxins tend to be more prevalent in seafood products, since they are produced by species with more estuarine habitats, allowing animals inhabiting these environments to tolerate a constant supply of toxins up to a limit that has not been well established (upstream). Species such as mussels and clams can steadily assimilate NODs (7.0–397 μg/kg in Sweden), which, even given the constant input of NODs, could promote, in some species, a more efficient detoxification dynamic (~70%, 72 h) [65,119,236,237].

The major turning point is represented by the STX-group, as these analogs have been described as being produced by some cyanobacterial genera (Table 1) [56,321,322,323], in addition to the dinoflagellate genera that produce them in marine environments (*Alexandrium* sp.) [241,324,325]. These toxins in a marine environment have a high prevalence in the food web (≥800 μg STX equiv/kg) due to annual blooms preventing total detoxification in some species of bivalves and gastropods [326,327,328]. Thus, to delimit the prevalence of toxins from the concentrations of cyanoHABs and HABs, it is necessary to carry out constant monitoring of both types of blooms, as well as analyze toxins from the cyanoHABs and HABs, since the incidence of these toxins in marine hydrobiological organisms may not be associated with HABs [144].

Likewise, most species in the sea stand out for having a high rate of bioconversion and detoxification of the STX-group (Phase I, CYP450, and Phase II via GST), which tend to broaden the toxin profile towards more toxic analogs [144,329]. However, it is possible that some species—when first exposed to cyanoHABs and/or HAB toxins—may experience harmful effects through oxidative stress or accumulation of the toxins in their tissues (feet), leading to paralysis and, consequently, stranding of the species on marine coasts [144,330,331].

Simultaneous events of filtration and accumulation of cyanotoxins and marine toxins have been confirmed by Anderson et al. [332], who detected the occurrence of MCs and domoic acid (DA) in estuaries in the U.S.A. This is due to blooms of *Pseudo-nitzschia* spp. contributing DA and MCs (intracellular + extracellular) from freshwater bodies, whose dynamic dispersion is favored by environmental factors (wind and temperature) [332].

Additionally, within the marine trophic network, secondary and tertiary consumers stand out significantly, most of which are fish, which, given the dynamics of the distribution and stability of cyanotoxins, make biomagnification determinations complex [333]. Ferrão-Filho et al. [138] established that in freshwater fish, the bioaccumulation factor is species-specific, reaching a value of < 3.5. This factor could be further simplified in a marine environment, where the possibility of bioaccumulation could be restricted and linked to NODs, given the habitat from which they originate [334]. Stewart et al. [271] detected different levels of NOD in different species representative of different levels of the food web, establishing that the bioaccumulation dynamics of the toxins was explained according to the chemistry of the toxin and the dynamics of the different types of fish species in the sea. Thus, fish such as *Platichthys flesus*, characterized by a mainly bottom-dwelling diet (crustaceans, mollusks, and annelids) have been found to possess NOD levels of >100 μg/kg, which are assimilated and absorbed mainly through browsing on the sandy bottom. *Salmo salar*, a carnivorous species whose diet is mostly composed of small fish, has a low capacity for the bioaccumulation of toxins, with concentrations of approximately <10 orders of magnitude, so according to the data, the part of the food web involving carnivorous fish is more related to the biodilution of toxins than to biomagnification, which is enhanced given their low exposure to toxins and the high metabolic capacity they possess [217,271,335]. Similarly, a general bioaccumulation trend of the different groups of cyanotoxins is prevalent in the digestive tissues, kidneys, gonads, and gills of fish, and to a lesser extent in their brains and muscle tissue, which can also exert damage to different organs according to the level of action that characterizes the group of toxins, leading to growth inhibition, behavioral changes, alterations in the antioxidant system, and mortality [3,138,336]. It should be noted that MCs have also been detected in seawater at concentrations ranging from 0.003 to 19.8 ng/L (Amvrakikos Gulf) [241] and that accumulation of the different groups of cyanotoxins has been identified in species such as *Anguilla* sp., *Clupea harengus*, *Osmerus eperlanus*, *Salmo salar*, and *Vieja* sp., among others (Table 4) [266,270,273,311].

### 3.3. Non-Traditional Vectors

The evaluation of cyanoHABs shows that most of the toxic events escape the logic of interaction within a trophic network, evidencing a direct route of exposure through the ingestion of cyanobacteria/cyanotoxins. Several articles have shown intoxications in sheep, cattle, dogs, fish, invertebrates, and other organisms, including higher plants [119,176,337,338].

The first scientific evidence of these poisoning cases was recorded in Australia, where a bloom of *Nodularia* sp. caused the death of sheep, horses, dogs, and pigs, and later, similar events were recorded in Europe (Table 4) [271]. Complementarily, in Canada and the U.S.A., blooms associated with *Microcystis aeruginosa* have caused the death of birds, in which concentrations of *Microcystis aeruginosa* were detected at ~6000 μg MCs/L [185]. While in Spain, the death of flamingos has been linked to blooms of *Microcystis aeruginosa*/*Anabaena flos-aquae*, in this case, the analyses correlated the deaths with the bioaccumulation process with respect to the levels of cyanotoxins detected in the water, corresponding to ~10 μg/mL MC, in relation to the content detected in the crops and livers of the birds (600 μg/mL and 440 μg/mL, respectively) [304].

#### Cyanotoxins in Crops

In view of this dynamic interaction of freshwater courses with cyanoHABs events, a high risk of using lentic and lotic waters for the development of hydroponic crops, or for the direct irrigation or sprinkling of soils intended for growing crops, such as lettuce, radishes, arugula, dill, parsley, alfalfa, broccoli, and cabbage, has been demonstrated. From this perspective, different studies have determined the bioaccumulation of CYN in different types of vegetables, such as spinach and lettuce, where the toxin has been detected in the roots and leaves [68,69,339,340].

Regarding crops, it has also been suggested that a constant supply of cyanotoxins (CYN, MCs, or NODs) can have toxic effects in plants that result in a decrease in seed germination, a reduction in photosynthetic yield, oxidative stress, and the alteration of plant growth and development (e.g., wheat, corn, peas, and lentils) [50,68,341,342]. In lettuce leaves, MC concentrations have been detected ranging from 0.094 to 2.487 μg MC-LR/g [343]. Concentrations exceeding 2.0 μg/g per day have also been reported, surpassing the regulatory levels established for drinking water [53]. Similarly, MCs are the most prevalent cyanotoxins detected in species such as apricot, grape, orchid, and plum, with toxicity levels varying between 0.1 and 177 μg/kg, depending on the species (Table 4) [344,345].

To understand this assimilation pathway, ref. [31] established that irrigation processes can have physical effects on cyanoHABs, which, combined with microorganism-mediated decomposition, gradually release intracellular cyanotoxins (MCs) into the environment. This allows the constant assimilation of toxins through the roots, stems, and leaves of plants, resulting in a clear bioaccumulation process. However, the direct application of MCs shows that soil microorganisms degrade these toxins, leading to a reduction in toxin availability and, consequently, a decrease in the rate of assimilation and accumulation by plants [31,341].

Thus, the consumption of stems, leaves, and edible crops can be considered an important vector, as their intake at concentrations > 0.04 μg MC/kg body weight/day may have harmful effects in extreme age groups (<10 or >65 years) [341]. The washing and rinsing process for certain plant species can help reduce cyanotoxin concentrations in leaves, but is less effective in stems. Nevertheless, it still lowers cyanotoxin levels to amounts that may not pose a risk to human health [2,61].

## 4. Human Exposure to Cyanotoxins

In freshwater bodies (lentic and lotic), cyanoHABs and their cyanotoxins are the main organisms that, in the last 20 years, have undergone an increase in their incidence and prevalence in various freshwater sources worldwide, becoming a public health problem [52] in many cases. Their negative effects in terms of human impacts mainly translate into interruptions in water supplies, closures of recreational areas, and poisoning [217,338,346,347].

The identification of cyanoHAB episodes is generally a straightforward process, as they can sometimes change the hue of the water source to a characteristic green color with the formation of dense scum masses, sometimes coupled with the production of an unpleasant odor [348]. However, to obtain an approximation of their real health impact, as a first step, it is necessary to identify the species responsible for the bloom, which in many cases may be mixed with toxic and non-toxic taxa, and simultaneously determine the densities of cyanobacteria in water bodies (cells/mL); however, the main problem and challenge lies in analyzing and quantifying the concentrations of cyanotoxins present in the water [304]. Consequently, multiple countries have adopted regulatory policies on the levels and densities of cyanobacteria in water, informing monitoring and alerts; in addition, some countries monitor the cyanotoxins in water according to reference values established by the WHO, while others monitor them according to their experiences, given that the risk is exposure to cyanotoxins (Table 3) [30,52,69,98].

Australia has set the maximum limit for recreational water use to a density of cyanobacteria of ≥40,000 cells/mL, and New Zealand has set the maximum limit for the consumption of drinking water to a cyanobacteria level of ≥1.0 μg/L of MCs [101].

The main routes of exposure to cyanotoxins tend to occur accidentally through the ingestion of water or contaminated food (water, saline, and vegetable vectors), the inhalation of contaminated particles (aerosols), or skin contact with contaminated water [3,52,349]. It is important to understand that exposure occurs because cyanotoxins are released into the water by different cyanoHAB degradation processes, among which photolysis, hydrolysis, and bacterial degradation stand out, and that according to the chemical properties of each group of cyanoHABs, they can persist actively for a long time following dilution [170,350].

The toxic symptoms related to cyanotoxins in humans are limited; evidence from proven cases allows us to establish that the initial symptoms associated with intoxication tend to be common to other clinical conditions (gastroenteritis, nausea, vomiting, diarrhea, allergies, etc. (Table 1), which somewhat hinders the first steps of anamnesis in hospitals or emergency centers [50].

Animal studies have determined that the main target tissue is the liver, tissue in which the first stage of detoxification occurs. In the case of MC-LR, the first stage involves an oxidation reaction that produces MC-LR-GSH (~60% in liver) to then favor cysteine conjugation and produce MC-LR-Cys, a metabolite that can promote the elimination of cyanotoxins through urine [231,289,351,352].

Among the most important and relevant cases of cyanotoxin exposure recorded to date is one that occurred in Brazil in 1996 (Caruaru), in which 116 patients from a dialysis center developed a clinical picture with symptoms that included headache, eye pain, blurred vision, nausea, and vomiting. Of the intoxicated patients, the conditions of ~100 were aggravated by the development of severe liver problems related to indicators of liver cell injury, including elevated serum aspartate aminotransferase (AST), alanine aminotransferase (ALT), and gamma-glutamyltransferase (GGT) concentrations. Seventy-six people died, and the presence of MCs and CYN in the water was identified as responsible for the problem. Subsequent analyses determined the presence of MCs in the serum of intoxicated patients at levels of up to 28.8 μg/L, while in asymptomatic patients, levels ranging from 0.2 to 0.96 μg MCs/L were detected. Thus, it could be established that the highest toxic effects and deaths that occurred in individuals were directly linked to intravenous exposure to MCs [101,127,276,277,278].

Subsequently, a similar event occurred in 2001 in Rio de Janeiro (Brazil) in which 44 patients from a dialysis center under similar procedures experienced identical clinical pictures [278].

Although what happened in Brazil is one of the best studied cases, in 1979, an outbreak in Australia led to the poisoning of 149 people, who experienced clinical symptoms that included fever, headache, vomiting, profuse bloody diarrhea, hepatomegaly, and renal damage. Subsequent epidemiological and ecological studies allowed the event to be associated with the presence of *Cylindrospermopsis raciborskii* in water bodies [338,353,354], and subsequently, it was possible to associate the poisoning with the presence of CYN in water [74,165]. CYN is characterized by inhibiting liver function as well as protein and glutathione synthesis, leading to cell death, and could explain this clinical picture.

To complement data on the concentration of toxins in water, Mchau [355] conducted a cross-sectional study using 432 people (~69% farmers) to determine the levels of exposure and risk of ingesting cyanotoxins through water consumption (Ukerewe in Mwanza, Tanzania). As a result, it was determined, through the UPLC-MS/MS technique, that the water samples collected showed toxicities ranging from 5.0 to 58.4 μg/L, while in the serum samples of the people, CYN-, NOD-, and MC-related analogs were identified. The concentration of CYN detected ranged from 0.02 to 0.15 ng/mL; concentrations of MC-LR, MC-RR, and dmMC-LR were detected at levels of 0.2–0.11 ng/mL (MC-RR < 0.02 ng/mL and dmMC-LR < 0.05 ng/mL), while the concentration of NOD was <0.05 ng/mL. These toxicities were found to be consistent with the regular symptoms presented by individuals (~50%), among which stomach upset, eye irritation, diarrhea (32%), vomiting (9%), and throat irritation (10%) were prominent [355]. However, to date, no incidents specifically attributed to NODs have been reported.

Among the groups most exposed to cyanoHABs and their toxins are children and adolescents, whose characteristics include smaller size (mass) and a greater tendency to ingest water and stay longer in the water during recreational activities (~2 h periods) [220]. In July 2002, a case was reported of several adolescents who, after playing in a pond with cyanobacteria (Wisconsin, USA), began to develop mild and severe clinical pictures, with common symptoms of nausea and diarrhea. The most severe symptoms occurred in the group that reported drinking the water; of those affected, one died after 48 h due to heart failure. Toxicological analyses of their feces and stomach contents were positive for the toxin anatoxin-a and the cyanobacterium *Anabaena flos-aquae*; despite this, chemical analyses were inconclusive in linking the death to cyanotoxins [356].

Another particular case was reported in 2007 in Argentina (Salto Grande), in which an adolescent, while riding a jet ski, fell into an area containing a bloom of *Microcystis* spp. Their exposure to the bloom lasted approximately 2 h before they were rescued. Four hours after exposure, the patient presented a clinical picture with characteristic symptoms of nausea, abdominal pain, and fever (duration between 48 and 72 h), which became more complex over the course of 3 days, with the presence of dyspnea and respiratory distress, at which point the patient was hospitalized with a diagnosis of atypical pneumonia. At this stage, renal failure, decreased platelets, increased leukocytes, and the development of hepatotoxicosis were also identified, with an evident alteration in markers related to liver damage (ALT, AST and γGT). Ecological evaluations and analyses related to the event identified the species *Microcystis aeruginosa* (~3080–4100 cells/mL) in the water body at concentrations ~48.6 μg/L contributed to by the MC-LR analog. Full recovery of the adolescent occurred 20 days after the incident [357].

Perhaps the most dramatic case associated with poisoning occurred in 2015 in Montevideo, Uruguay. A few hours after recreational activities on a beach in the area, a family began to present gastrointestinal symptoms, which for one of them (a 20-year-old) began to be more limiting (diarrhea, vomiting, fatigue, and jaundice). These symptoms aggravated the patient’s situation, which, according to a laboratory analysis, evidenced a picture of anemia, coagulopathy, and increased serum levels of ammonium, alanine aminotransferase (ALT), aspartate aminotransferase (AST), and bilirubin. After five days, the patient began to present acute liver failure, which triggered autoimmune hepatitis type II (AH-II). Their clinical picture finally led to liver transplantation surgery. In parallel, water samples from the area determined MC levels between 2900 and 8200 μg/L. An analysis of the MCs in the liver extracted from the patient identified the presence of MC-LR and [D-Leu1]MC-LR at concentrations of 2.4 and 75.4 ng/g tissue. These results, when linked to ecological data from the area, estimated that the person may have ingested ~1.78 L of the water [358].

Another relevant pathway for cyanotoxin ingestion is through food webs, which increases the risk of human exposure through the consumption of vectors (bivalves or fish) from water bodies with high prevalence of cyanoHABs or cyanoHABs and/or cyanotoxin receptors. Thus, exposure from fish consumption is critical, as fish have been shown to accumulate toxins in their livers and may eventually accumulate them in their muscles; bivalves can also accumulate cyanotoxins for periods longer than 6 months, with cyanotoxins possessing the ability to be biotransformed into more toxic analogs, which could result in lower clearance rates for certain analogs [304]. One study identified MCs in the serum of fishermen, probably related to the ingestion of MCs through water and the consumption of contaminated fish. Alterations in blood biochemical parameters (ALT, AST, LDH, and ALP) related to hepatocellular damage correlated with likely MC infection through water consumption at levels of ~1.31 μg MC-LR per day (2 L per day), and that through fish consumption (intake of ~100–300 g per day) could correspond to ~0.86–2.57 μg MC-LR, exceeding the total TDI of 2.4 μg for an adult of ~60 kg [232,289].

Another route of exposure occurs through the inhalation of cyanotoxins produced by the aerosol produced in waves. However, studies conducted involving the collection and analysis of aerosols over 4, 12, and 24 h have shown that their concentrations do not constitute a direct danger to people, with risks limited only to extreme age groups or to people with prevalent respiratory diseases (asthma) [2].

Empirical data related to cyanotoxin intoxications are very limited, and only those of greater consequence have adequate data traceability. Bloch et al. [359] conducted an epidemiological study on cases related to cyanoHABs exposure in the U.S.A. The data showed that of the total cases reported of environmental exposure in watercourses, only ~20% were related to cyanoHABs. Most of the cases were characterized by acute symptoms, among which the most frequently reported clinical symptoms were diarrhea, vomiting, nausea, skin rash, and abdominal pain, and in some cases, symptoms such as fever, throat irritation, and cough were reported. In those cases, linked to water ingestion, the most common symptoms were diarrhea, vomiting, abdominal pain, and nausea. Of the total number of cases analyzed, only 2% presented severe symptoms, with no evidence of death [359]. However, other studies have reflected significant cases linking incidents of cyanoHABs to acute and chronic gastrointestinal diseases through exposure [360]. It should be noted that MCs do not enter the human body through dermal exposure due to the size of the molecules and their chemical nature, in addition to the fact that their clinical effects are associated with the ingestion or aspiration of water or food containing cyanobacterial cells and/or cyanotoxins, which are then distributed to target tissues through active transport mechanisms. This possibility can be extrapolated to NODs, considering that they share a similar chemical structure to MCs, which allows them to have the same level of toxic action, although it is important to point out that there are not enough epidemiological data linked to human cases [304,361].

From a chronic point of view, epidemiological studies (~80 years) have established a direct relationship between the high prevalence of primary liver cancer and the ingestion of water contaminated with MCs in China and Croatia [92,362].

According to the data presented, the most common routes of exposure may be through dermal exposure; inhalation in recreational (sports), professional (fishing), or domestic (showers) activities; ingestion through vectors; or the absorption of cells and toxins ingested with water, generally unintentionally and parenterally (accidental) [53]. The level of severity of intoxications will largely depend on the route of intoxication, toxicity levels, and duration of exposure, which is complemented by physiological capacities that link the absorption, detoxification, and excretion capacity of cyanotoxins [61].

## 5. Risks of cyanoHABs

At present, toxic cyanobacteria are recognized as the group of organisms primarily responsible for cyanoHAB-related events. They are becoming increasingly complex and persistent, producing longer bloom periods, with the consequent transfer of multiple types of cyanotoxins to the food web, leading to a wide range of public health effects [363].

Cyanotoxins correspond to a set of secondary metabolites produced by different genera and species of cyanobacteria, which, through interaction and assimilation with the trophic network, can contaminate different species. Cyanotoxin exposure via these routes has been linked to various hepatotoxic, cytotoxic, and neurotoxic effects in humans, resulting in acute and chronic poisoning [304,332]. The increased incidence in areas of Central and South America has established important challenges in the areas of taxon classification, laboratory cultures, and the determination of strain toxicity, whereby cyanoHAB events and the cyanotoxins involved represent a potential risk to human and animal health, given their wide range of toxicities arising from the different toxin groups, in which the risks of chronic exposure to human health are unknown in some groups (STXs) [50,232,245,364].

To determine the environmental and public health risks, it is necessary to evaluate the concentrations of cyanotoxins observed in the environment and the toxicity levels established in different experimental studies. This allows the risks associated with the different taxa of cyanoHABs to be assessed, including the evaluation of the variability of naturally synthesized analogs and those linked to biotransformation processes in vectors coming from different aquatic ecosystems and trophic levels. This makes it possible to assess the risk of simultaneous cyanoHAB events involving the possibility of exposure to a mix of analogs which could lead to the potentiation of effects by exceeding toxicity thresholds, making them a risk to public health [16,50,52,204,365].

To understand and avoid these circumstances, international organizations have created and proposed norms that establish limits for exposure to cyanoHABs and cyanotoxins [53,103], which are referential and which, despite the exposure and seafood poisoning in Central and South America, are not yet considered within sanitary regulatory norms.

From this perspective, some countries use guideline reference values for cyanobacterial biomass (low risk < 20,000; moderate 20,000–100,000; high 100,000–10,000,000; very high > 10,000,000 cells/mL); pigment concentrations (low risk < 10; moderate 10–50; high 50–5000; very high > 5000 chlorophyll-α); and cyanotoxin concentrations (low risk < 10; moderate 10–20; high 20–2000; very high > 2000 μg/L MC-LR) [30,53]. Densities of 11,500 cells m/L of *Microcystis aeruginosa* can produce MC-LR toxicity of 2.3 μg/L [201].

In order to fill the gaps and be able to respond adequately to benchmark indicators, national health agencies must establish and have the necessary tools to allow for adequate risk management; among them, the following stand out: the technical and professional capacity for the taxonomic identification of cyanobacteria (toxic and non-toxic); the analytical capacity to identify cyanotoxin groups in freshwater bodies and estuaries (MCs, ATXs, CYNs, STXs, and NODs); the capacity to develop detection and quantification methods in different biological matrices to determine the level of bioaccumulation and/or biomagnification in different species; the acquisition of analytical standards for the identification of groups of cyanotoxins; the constant training of work teams and dissemination of technical data to decision makers (authorities, mayors, governments, medical services, etc.) [61].

From the point of view of effects, toxicity assessment involves an analysis of the amounts or doses of a chemical substance ingested by a person or an animal that causes adverse health effects [304]. Thus, the acute reference dose (ARfD) is defined as an estimate of the amount of a substance present in food or drinking water that can be ingested in a period of 24 h or less, without an appreciable risk to human health (expressed as a function of body weight), and which derives from the no-observable-adverse-effect level (NOAEL) [142,317]. Meanwhile, the total daily intake (TDI) allows the risk to be assessed through the average food intake over a lifetime [317].

Thus, the WHO has established a TDI for MC-LR < 0.04 μg per kg body weight, considering the reference limit in drinking water of MC-LR < 1 μg/L (NOAEL MC-LR 40 μg/kg/day). Based on this, it has been proposed that if a person in recreational swimming activities ingests ~100 mL of water, the exposure limit corresponds to 24 μg of MC-LR per liter of bathing water (Table 2) [52,87,95].

Additionally, a TDI for MCs in seafood of 24 μg/kg wet weight has been established based on a 60 kg person consuming ~100 g of seafood per day. For fish consumption, the established limits are based on the consumption and mass of children and an adults, with the reference values being 4.0 μg/kg and 8.0 μg/kg, respectively [87,232,302,303].

In relation to the STX-group, the EU has established a limit for this group of toxins in bivalve mollusks corresponding to 800 μg STX-2HCl equiv/kg of meat. This limit is intended to protect consumers from acute risks; in fact, 144–304 μg of STX/person has been found to produce acute intoxication in humans. Toxicities > 450 μg of STX/person can produce severe intoxications, and >1000 μg of STX/person leads to death [157]. These data cannot be used to assess chronic risks from repeated exposure to STX, as no information is available [157].

However, several studies consider that the toxicities of some groups of cyanotoxins may underestimate the risk, since calculations have been made on the basis of some toxic analogs, and it has been found that the ratio of free and covalently bound toxins to tissues may vary over time (MCs) so that the toxic capacity of the analogs during the digestion process is increased [70,366,367]. Likewise, the risk of human intoxication may increase, especially when people consume freshwater or saltwater vectors (estuaries) contaminated with cyanotoxins. A relevant point is that cooking processes do not destroy or denature cyanotoxins, but can promote the conversion of analogs, increasing the toxin profile, and some post-cooking toxic groups may increase in toxicity by being free from tissues (MCs) [12,368]. Hence, it is important to implement frequent monitoring of cyanobacteria and cyanotoxins in water reservoirs [119].

Currently, many countries have not implemented standards or the WHO guidelines, and warnings about cyanoHABs are only carried out when a bloom is evident and has a visual impact or impacts tourism, but without reporting on toxicities and toxin profiles involving water bodies [361].

Eutrophication and high climatic variability are related to cyanoHABs, promoting their frequency and sometimes determining profiles from increasingly toxic taxa in freshwater bodies and sometimes producing subacute/subchronic exposure through recreational activities [61]. Additionally, high water flow (lotic zones) allows the expansion of cyanoHABs and cyanotoxins towards estuarine and marine areas, which favors the dispersion and consequent accumulation of cyanotoxins in marine resources, which can have serious effects on public health due to intoxication through vector consumption [52,369,370].

Thus, for an adequate evaluation of water bodies intended for recreational activities (fishing and bathing), it is necessary to carry out regular monitoring, which will generate relevant information on the dynamics of the cyanobacterial community and its toxicity (including types of toxins and the relationships between analytes), which is complemented by relating them to the trophic status of the water bodies (Universal, Carlson and TRIX). Critical variables such as a chlorophyll concentration > 1.0 μg/L and the identification of potentially toxic cyanobacteria at densities > 20,000 cells/mL can be considered the first step in establishing a monitoring level in affected areas. Densities of 20,000 cells/mL, of *Microcystis aeruginosa* can yield 2–4 μg/L or up to 10 μg/L in the case of particularly toxic taxa [61,365]. In terms of risk, in this instance, monitoring should be complemented with molecular techniques that allow the toxic potential of cyanobacteria to be defined (duration of 4 h) along with an ELISA test for the determination of toxicities (duration of 2 h), since at this point, the consideration only of cell counts can cause an overestimation when the proportion of toxic versus non-toxic individuals is low; likewise, toxicity can be underestimated if the bloom is in the senescence phase, the period in which toxicity is higher in water. From this point, communication with local health authorities, tourism authorities, and decision makers is necessary for future actions to be taken.

**Figure 4:**
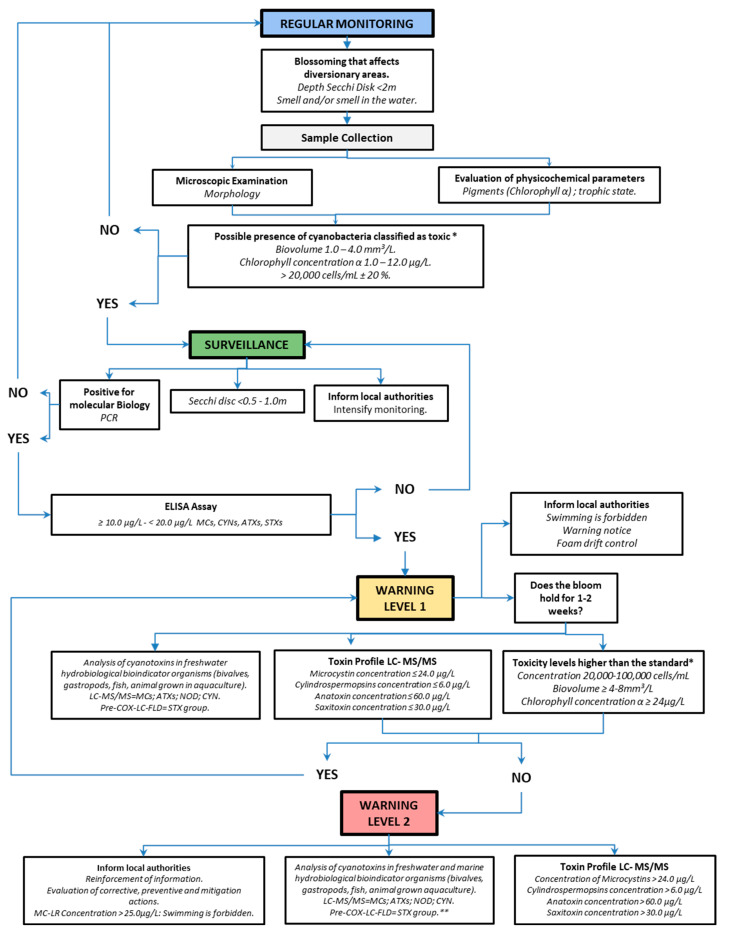
Framework for monitoring and managing cyanobacteria and cyanotoxins in recreational water bodies. * WHO, 2021. ** European Union, 2017.

Persistence of the bloom (1–2 weeks) can produce densities between 20,000 and 100,000 cells/mL (90 μg/L of Chlorophyll-a), which has been associated with ocular mimicry in bathers, and the maximum limit in the case of *Microcystis* spp. can generate 20 μg/L of toxin [363]. Therefore, it is essential to complement the data with LC-MS/MS analysis to determine the profile and concentrations of cyanotoxins in cells and water, which, according to the established levels, could warrant a level 1 alert. Worsening of these data (cyanobacterial density, chlorophyll concentration, toxicities in water) warrants a level 2 alert, which is established by complementing the analyses in vectors obtained through sports activities and in marine species of habitual consumption or commercial interest (Figure 4). For these two alert levels, relevant decisions are required, such as the precautionary closure of recreational areas, the prohibition of bathing (people and pets) and of vector extraction (fish and bivalves), and the prohibition of water use for consumption or use for irrigation in agricultural production and, eventually, the consumption of seafood products [12]. Lotic synthems can transfer cyanoHABs and cyanotoxins to marine areas, where species characteristic of the ecosystem can leach and accumulate cyanotoxins (MCs, NODs, and STXs) in their tissues [49].

Thus, in order to establish a logical framework, it is essential to identify biomarkers of exposure in order to conduct appropriate biomonitoring studies, which will allow for constant re-evaluation of the risks and hazards [69].

## 6. Discussion

Aquatic ecosystems are made up of photosynthetic organisms such as macrophytes, microalgae, and benthic/planktonic cyanobacteria, which fix carbon, produce oxygen, and form the basis of food webs [371,372]. The interaction of multiple environmental factors contributes to and favors the growth of different microalgae in the sea, lakes, and rivers. These factors correspond to light intensity, water temperature, pH, carbon dioxide concentration, nutrient availability (nitrogen, phosphorus, iron, and molybdenum), the physical characteristics of the water (shape and depth), the stability of the water column, water flow (rivers), and the structure and function of aquatic ecosystems [143,373].

CyanoHABs correspond to natural events; however, in the last 60 years, as a consequence of human development and the increase in agricultural, urban, and industrial activities, there have been important changes in the environments of lakes and rivers (eutrophication) leading to a higher incidence and prevalence of cyanoHABs worldwide [57,374], which has been attributed to the process of climate change [163,375,376]. It has therefore been proposed that a synergistic interaction between increased eutrophication and climate change has led to the development of new events associated with cyanoHABs in lentic and lotic zones [57,377]. The Intergovernmental Panel on Climate Change (2019) [378] has established that the incidence of harmful algal blooms, their toxicity, and the consequent risk to natural and human systems will continue to increase in direct relation to global warming, which will reach 1.5 °C over the next 20 years, in addition to the increase in CO_2_ emissions in the 21st century [7,378].

Rigosi et al. [29], evaluating pollutants that contribute to the determination of the trophic state in water bodies, determined that high biomasses of cyanobacteria are characteristic of oligotrophic water bodies, with nutrient input being the major factor; in mesotrophic lakes, the temperature factor showed a more direct relationship with an increase in bloom. In eutrophic and hypereutrophic lakes, a significant interaction between nutrients and temperature was observed [29].

By evaluating isolated components, it can be established that microalgae show a strategy of adaptation to extreme temperature changes. *Microcystis aeruginosa* exhibits seasonal succession with temperature changes, producing blooms in summer (ideal temperature ~20 to 25 °C); in autumn, it sinks in the water, and in winter, it tends to inhabit the surface layer of the sediments without losing viability, progressively returning to the system through the water column in spring by regulating its gas vesicles, which allows it to float (benthic–planktonic phase) [28,379,380]. This process is also related to *mcyB* gene expression and MC content, since high temperatures (>20 °C) tend to increase MC production, while low (<4 °C) and very high (>35 °C) temperatures decrease it [46,381,382].

Nevertheless, cyanoHAB events occur in the face of a biotic–abiotic interaction, in which the stoichiometric relationship is difficult to establish and forecast. Thus, climatic variability in terms of rainfall (intense or intermittent rainfall) may lead to the interruption of blooms in summer periods, after which proliferation of the same species or other potentially toxic species in the ecosystem is possible. This raises the possibility that for certain communities, the frequency, duration, and intensity of cyanoHABs may increase in certain water bodies [6,52].

From an ecological and environmental perspective, the effects of current and probably continuous climate variability in the long term will lead us to a scenario of climate change that includes modifications in the patterns of precipitation and temperatures in different regions of the world. Thus, climate change and human impact (nutrient inputs) may trigger future variations in the magnitude, frequency, and length of the period of cyanoHABs [383,384,385,386]. Therefore, cyanoHABs are a problem in aquatic ecosystems and, subsequently, through different exposure pathways, can alter public health [261,387,388,389].

This represents a major risk scenario from a marine point of view. Several studies have already established the real possibility that, through currents, cyanoHABs can be distributed from lotic ecosystems (MCs) to estuaries and/or from estuaries (NODs) to the sea. During this pathway, the physicochemical interactions of the aquatic environment favor cell lysis, which allows cyanotoxins to efficiently dispose of the medium, which can be filtered, accumulated, biotransformed, and removed from highly efficient organisms in the sea (mussels). This is a major challenge, since cyanotoxins are not included in permanent monitoring programs for their detection and quantification, leading to the possibility of intoxication in people, mostly in extreme age groups (children and the elderly), or generating subtoxic levels that lead to the chronic consumption of some groups of cyanotoxins, especially those classified as possibly carcinogenic [49,229].

In addition, the high chemical variability among the different groups of cyanotoxins brings an important challenge in the identification and quantification of the different analogs and especially of the new chemical variants that are detected and characterized every year from different geographical areas, which is also related to identifying and properly tabulating the cyanobacteria producing-groups of cyanotoxins and understanding their stages of toxin production in relation to nutrient inputs, temperature, and/or rainfall [318,390].

In recent years, the human population has experienced rapid growth, especially post-pandemic, in which there has been a migration of the urban population to rural areas, generating a drastic change in land use and demographically modifying areas that were historically used for agriculture. This has resulted in a considerable impact on lake areas, where water use has been altered, leading to the implementation of new irrigation systems, which have begun to generate significant inputs of anthropogenic organic waste, contributing to and favoring the incidence of cyanoHABs [228,391].

The different scenarios studied in the literature clearly demonstrate that cyanoHABs will intensify worldwide, creating a major challenge for water resources, tourism, health, and political administrations. The vast majority of countries do not consider the option of implementing the Guidelines for drinking-water quality and Guidelines for safe recreational water environments [53,392], which translates into a major problem in the generation of databases on the prevalence and incidence of cyanoHABs and, at the same time, represents a challenge for stakeholders in the face of events of great magnitude and impact involving the ingestion of cyanotoxins through drinking water or the consumption of contaminated shellfish and/or fish [229,393]. Therefore, it is important that in those countries where cyanoHAB events are increasing in incidence and prevalence, stakeholders establish international agreements in their respective countries and complementarily allocate and/or manage economic resources to increase the technical capabilities of experts for the proper identification of cyanoHAB-producing taxa through microscopic (Utermöhl technique) and molecular (RT-qPCR technique) techniques. Based on standardized procedures, records can be made to establish the place and time in which blooms occur, allowing relationships to be established between identified species and the densities, pigment types, biovolume, and trophic status of water bodies [385]. These involve bioconversion processes of cyanotoxins through various vectors (fish and shellfish) which, together with recreational activities as well as drinking water consumption, can cause significant public health problems. Therefore, the WHO has proposed limits for the different groups of cyanotoxins for both recreational activities and drinking water consumption (Table 2) [50,198].

The implementation of these technical proposals will help to direct preventive monitoring towards regular monitoring, which will allow the development of a database and, thus, through this information, the adequate evaluation of health problems, the mitigation proposals, and the comparison of harmonized data between countries. In this way, regional or global decision making is favored, which can be translated into optimized guidelines or international standards for each of the groups of cyanotoxins that are already known or those yet to be identified.

## 7. Conclusions and Future Challenges

CyanoHABs constitute an increasingly relevant threat to freshwater aquatic ecosystems, where environmental (abiotic–biotic) and anthropogenic interactions, such as global warming and the eutrophication of water bodies, provide the ideal scenario for an increase in their incidence and prevalence on a global level, through alterations in the community and diversity of microorganisms with a concomitant deficiency in water quality.

Direct (water) or indirect (vectors) contact with cyanoHABs/cyanotoxins causes adverse reactions, organ damage, and even death in humans. The cases produced and/or registered in relation to human health show the deficiency in high-quality and timely information for health professionals, generating a barrier to taking quick and adequate action when facing serious poisoning situations. Anatomopathological and epidemiological evaluations have helped us to understand this problem, reflecting the lack of information and training on the subject at this level.

Once sustainable monitoring programs are established, those with responsibility for maintaining waterbodies (e.g., for use as drinking water sources or for recreation) could use the historical results from these programs to develop guidelines for acceptable levels of cyanotoxins.

## Figures and Tables

**Table 4:** Occurrence of cyanotoxins in food web transfer.

Typical Species					
Species		Cyanotoxins	Toxin Accumulation	Method of Detection	References
**Bivalvia**					
**Freshwater**					
Bivalve	*Alathyria pertexta*	CYN	130.0–560.0 μg CYN/kg	HPLC	[229]
	*Anodonta cygnea*	CYN	2.9–61.5 μg/g DW	HPLC	[230]
	*Anodonta woodiana*	MC	12.6 μg/kg DW	HPLC	[231]
	*Cristaria plicata*	MC-LR/YR/RR	0.07 μg/g DW	LC-MS/MS	[232]
	*Unio douglasiae*	MC	420.0 μg/kg DW	HPLC	[231]
	*Corbicula fluminea*	STX	0.4–0.6 μg/g DW	HPLC	[233]
**Seawater**					
Bivalve	*Alanthyria condola*	STX	0.8–6.2 μg/g DW	HPLC	[234]
	*Macoma balthica*	NOD	0.16–30.0 μg/g DW	LC-ESI-MS	[235]
	*Macoma balthica*	NOD	320.0 μg/kg DW	HPLC-DAD	[236]
	*Magallana gigas*	NOD	24.1–397.3 μg/kg	LC-MS/MS	[237]
	*Mytilus edulis*	NOD	0.28–13.8 μg/g DW	LC-ESI-MS	[236]
	*Mytilus edulis*	NOD	2200.0 μg/kg DW	ELISA-LC-MS	[238]
	*Mytilus edulis*	NOD	400.0–1100.0 μg/kg DW	LC-MS/MS	[239]
	*Mytilus galloprovinciales*	MC-LR/YR/RR	0.7–53.9 ng/g	ELISA/UHPLC-HRMS	[71]
	*Mytilus galloprovinciales*	ATX	6.6 ng/g DW	HPLC	[240]
	*Mytilus galloprovinciales*	MC-LR	45.0–141.5 ng/g	ELISA	[241]
	*Mytilus trossulus*	MC-LR/LA/LW	1.9–32.3 μg/kg DW	LC-MS/MS	[242]
**Gastropoda**					
**Freshwater**					
Crayfish	*Paranephrops planifrons*	NOD	9.7–225.3 μg/kg WW	LC-MS	[243]
Lobster	*Cherax quadricarinatus*	CYN	0.54–4.3 μg/g	HPLC	[244]
Snail	*Bellamya aeruginosa*	MC-LR	6.61 μg/g DW	LC-MS/MS	[245]
	*Bellamya aeruginosa*	MC-LR/RR	1.06–7.42 μg/g DW	LC-MS	[246]
	*Helisoma trivolvis*	MC	37.0 μg/g DW	HPLC	[231]
	*Hippeutis complanatus*	MC	1223.26 ng/g FM	HPLC	[247]
	*Lymnaea stagnalis*	MC-LR	0.26 μg/g DW	ELISA	[248]
	*Margaria melanoides*	MC	0.40 μg/g DW	ELISA	[249]
	*Melanoides tuberculata*	CYN	ND–250.0 μg/g DW	HPLC	[240]
	*Physa acuta*	MC	1325.45 ng/g FM	HPLC	[250]
	*Physa gyrina*	MC	129.0 μg/g DW	HPLC	[231]
	*Planorbis planorbis*	MC	548.33 ng/g FM	HPLC	[250]
	*Pomacea patula catemacensis*	CYN	3.35 ng/g	LC-MS/MS	[251]
	*Pomacea patula catemacensis*	STXs	1.04–21.34 ng/g	ELISA	[251]
	*Sinotaia histrica*	MC-LR	9.03 μg/g DW	HPLC	[252]
Shrimp	*Mysis relicta (Decapoda)*	NOD	0.5–0.74 μg/g DW	ELISA/PP1	[253]
**Seawater**					
Crayfish	*Callinectes sapidus*	MC	105.0 μg/L	ELISA	[254]
	*Cherax quadricarinatus*	CYN	0.9–4.3 μg/g DW	HPLC	[244]
Shrimp	*Macrobrachium nipponensis*	MC-LR	0.24 μg/g DW	LC-MS/MS	[245]
Snail	*Vaughtia fenestrata*	CYN	0.8 ng/g	ELISA	[255]
Prawn	*Penaeus monodon*	NOD	6.0–80.0 μg/kg DW	ELISA-LC-MS	[256]
**Actinopterygii**					
**Freshwater**					
Fish	*Anguilla australis*	NOD	24.0 μg/kg	LC-MS/MS	[250]
	*Anguilla reinhardtii*	NOD	58.6 μg/kg DW (liver)	LC-MS/MS	[251]
	*Bramocharax caballeroi*	CYN	0.81 ng/g	ELISA	[255]
	*Ctenopharyngodon idellus*	MC-LR/YR/RR	0.04 μg/g DW	LC-MS/MS	[246]
	*Carassius auratus*	MC-LR	150.0 ng/g DW	LC-ESI-MS	[257]
	*Cyprinus carpio*	ATX-a	30.0 ng/g DW	GC/MS	[258]
	*Cyprinus carpio*	MC-LR/YR/RR	0.10 μg/g DW	LC-MS/MS	[257]
	*Gasterosteus aculeatus* L.	NOD	2.8–700.0 μg/kg	LC-MS/MS	[259]
	*Geophagus brasiliensis*	STX	1.22–1.97 μg STX equiv/100 g	HPLC-FLD	[260]
	*Hypophthalichthys molitrix*	MC	1.16–17.8 μg/kg DW	HPLC	[231]
	*Hypophthalmichthys molitrix*	MC-LR/YR/RR	0.08 μg/g DW	LC-MS/MS	[257]
	*Lates niloticus*	MC-LR/YR/RR/LA	0.7 μg/kg DW	LC-MS/MS	[261]
	*Melanotaenia eachamensis*	CYN	1.2 μg/g DW	HPLC	[262]
	*Oncorhynchus mykiss*	MC-LR	90.66 ng/g DW	LC-MS/MS	[263]
	*Oreochromis niloticus*	MC-LR/YR/RR/LA	0.6–15 μg/kg DW	LC-MS/MS	[261]
	*Oreochromis niloticus*	CYN	0.417 µg/g DW	ELISA/HPLC	[264]
	*Perca flavescens*	MC	130.0 µg/g DW	ELISA	[265]
	*Perca fluviatilis* L.	MCs	50.0 µg/g	HPLC-UV	[266]
	*Rastrineobola argentea*	MC-LR/YR/RR/LA	23.4 μg/kg DW	LC-MS/MS	[261]
	*Rutilus rutilus*	NOD	900.0 μg/kg DW	ELISA/LC-MS/MS	[267]
	*Silurus glanis*	MC-RR	0.14 μg/g DW (muscle)	HPLC	[259]
	*Salmo trutta*	NOD	125.0 μg/kg DW	ELISA	[268]
	*Tilapia rendalli*	MC	2.9–67.8 µg/g PS	HPLC/ELISA	[269]
**Seawater**					
Fish	*Ariosoma mellissii*	MC-LR	28.1 μg/kg DW	ELISA	[270]
	*Clupea harengus*	NOD	6.5 μg/kg DW	ELISA	[259]
	*Clupea harengus membras* L.	NOD	0.0–90.0 μg/kg	LC-MS/MS	[252]
	*Gadus morhua*	NOD	0.05 µg/g DW	LC-MS/MS	[238]
	*Melanotaenia eachamensis*	CYN	1.2 µg/g DW	HPLC	[244]
	*Mugil cephalus*	NOD	32.3–56.8 μg/kg DW	LC-MS/MS	[271]
	*Osmerus eperlanus*	MCs	874.0 µg/g DW	HPLC-DAD	[266]
	*Platichthys flesus*	NOD	1.0 μg/kg DW	ELISA	[272]
	*Platichthys flesus*	NOD	100.0–600.0 μg/kg WW	LC-MS/MALDI-TOF-MS	[273]
	*Platichthys flesus*	NOD	22.0–557.0 μg/kg	HPLC	[274]
	*Salmon salar*	NOD	5.0–10.0 μg/kg	ELISA/LC-MS/MS	[252]
	*Vieja* sp.	CYN	0.42 ng/g	ELISA	[255]
**Atypical Species**					
**Species**		**Cyanotoxins**	**Toxin accumulation**	**Method of Detection**	**References**
**Humans**					
Human	Homo sapiens-sapiens.	MC	2.03 μg daily MC intake	HPLC	[275]
	Homo sapiens-sapiens.	MC-LR/YR/RR	2.2–3.9 μg daily MC intake	LC-MS/MS	[232]
	Homo sapiens-sapiens.	MC	2.2 ng/mL	ELISA	[276]
	Homo sapiens-sapiens.	MC	7.1–31.4 ng/mL	LC-MS/MS	[277]
	Homo sapiens-sapiens.	MC	0.16–0.96 ng/mL	ELISA	[278]
**Mammals**					
Cow	*Aberdeen angus*	MC-LR	7100.0 μg/L (rumen)	LC-MS/MS	[279]
	*Bos Taurus*	MC	5.7 ± 0.5 mg/L	ELISA	[280]
Buffalo	*Bison bison*	MC	9.7 ± 1.4 mg/L	ELISA	[237]
Deer	*Capreolus capreolus*	MC-YR/LR/RR	1.36 μg equiv MC-LR/g	LC-MS	[281]
Dog	*Canis lupus familiaris*	MC	100.0 mg/g DW	ELISA–LC-MS/MS	[282]
	*Flat-coat Retriever*	ATX	1.04 µg/g	LC-HRMS	[283]
	*Golden retriever*	dihydroanatoxin-a (dhATX)	974.88 ng/g DW	LC-HRMS	[284]
	*Labrador*	Homo-ATX-a	9.5 μg/g DW	LC-MS/MS	[285]
	*Yorkshire terrier*	ATX-a	0.6 mg/g (liver)	HPLC-UV-LC-MS/MS	[286]
	*Canis lupus familiaris*	ATX	357.0–785.0 mg/kg	LC-HRMS	[287]
Dolphin	*Tursiops truncatus*	MC/NOD	14.3 ± 5.6 ng/g DW	ELISA-LC-MS/MS	[288]
Pig	*Sus scrofa domesticus*	MC-LR	26.4 μg/g DW	LC-MS/MS	[289]
Sea Otter	*Enhydra lutris*	MC-RR	1.36–104.46 μg/g	LC-MS/MS	[106]
**Amphibians**					
Bufo marinus	*Rhinella marina*	CYN	895.0 µg free-CYN/kg FW	LC-MS/MS	[290]
Bullfrog	*Lithobates catesbeianus*	MC	1 μg/L	ELISA	[291]
Rana eperotica	*Pelophylax epeiroticus*	MC	0.26–0.47 μg/g DW	PP2/ELISA	[290]
**Reptiles**					
Turtle	*Emys orbicularis*	MC-LR	0.001–37.2 μg/g DW	PP2A/Limieux-GC-MS	[292]
	*Mauremys leprosa*	MC-LR, -RR, -YR	0.02–1.193 μg/g DW	PP2A/Limieux-GC-MS	[292]
	*Pelodiscus sinensis*	MC	0.011–0.021 μg/g DW	LC-ESI-MS	[257]
**Birds**					
Black-crowned night heron	*Nycticorax nycticorax*	MCs	10.0 ng/g DW gonad	LC-ESI-MS	[257]
Chicken	*Gallus gallus domesticus*	MC-LR	20.0 μg/kg	Bioassay	[293]
Domestic duck	*Anas platyrhynchos*	MC	0.031 μg/g DW (liver)	HPLC	[231]
	*Anas platyrhynchos*	MCs	15.0 ng/g DW muscle	LC-ESI-MS	[257]
Duck	*Somateria mollissima*	NOD	3.0–180.0 μg/kg DW	LC-MS/MALDI-TOF-MS	[294]
Flamingo	*Phoeniconaias minor*	ATX-a	7.62 μg/g DW	LC-MS/MS	[295]
	*Phoenicopterusruber*	MC	625.0 μg equiv MC-LR/mL	ELISA	[296]
**Plant Kingdom**					
Apricot	*Prunus armeniaca* L.	MC	7.20 ± 0.85 μg/kg DW	ELISA	[232]
Grape	*Vitis vinifera* L.	MC	0.10 ± 0.02 μg/kg DW	ELISA	[232]
Lettuce	*Lactuca sativa* L.	MC	8.31–177.8 μg/kg FW	LC-MS/MS	[233]
Plum	*Prunus domestica* L.	MC	7.17 ± 0.39 μg/kg DW	ELISA	[232]
Aquatic plant	*Ceratophyllum demersum*	MC	0.1–0.2 µg/L	HPLC	[297]
	*Myriophyllum spicatum*	MC	0.5 µg/L	HPLC	[297]
**Chordata**					
Tunicate	*Microcosmus sabatieri*	ATX-a	193.7–1240.2 μg/kg	LC-MS/MS	[298]

DW: dry weight. WW: wet weight. FW: fresh weight. FM: fresh muscle.

## Data Availability

No new data were created.

## References

[1] Cirés S., Casero M.C., Quesada A. (2017). Toxicity at the Edge of Life: A Review on Cyanobacterial Toxins from Extreme Environments. Mar. Drugs.

[2] Sanseverino I., Conduto António D., Loos R., Lettieri T. (2017). Cyanotoxins: Methods and Approaches for Their Analysis and Detection.

[3] Jacinavicius F.R., Geraldes V., Fernandes K.A., Crnkovic C.M., Gama W.A., Pinto E. (2023). Toxicological effects of cyanobacterial metabolites on zebrafish larval development. Harmful Algae.

[4] Codd G.A., Testai E., Funari E., Svirev Z. (2020). Cyanobacteria, cyanotoxins, and human health. Water Treatment for Purification from Cyanobacteria and Cyanotoxins.

[5] Reinl K.L., Brookes J.D., Carey C.C., Harris T.D., Ibelings B.W., Morales-Williams A.M., de Senerpont Domis L.N., Atkins K.S., Isles P.D.F., Mesman J.P. (2021). Cyanobacterial blooms in oligotrophic lakes: Shifting the high-nutrient paradigm. Freshw. Biol..

[6] Harris T.D., Reinl K.L., Azarderakhsh M., Berger S.A., Castro Berman M., Bizic M., Bhattacharya R., Burnet S.H., Cianci-Gaskill J.A., de Senerpont Domis L.N. (2024). What makes a cyanobacterial bloom disappear? A review of the abiotic and biotic cyanobacterial bloom loss factors. Harmful Algae.

[7] Huertas Romera M.J., Mallén Ponce M.J. (2021). Dark side of cyanobacteria: Searching for strategies to control blooms. Microb. Biotechnol..

[8] Bouteiller P., Lance E., Guérin T., Biré R. (2022). Analysis of total-forms of cyanotoxins microcystins in biological matrices: A methodological review. Toxins.

[9] Facey J.A., Apte S.C., Mitrovic S.M. (2019). A review of the effect of trace metals on freshwater cyanobacterial growth and toxin production. Toxins.

[10] Burford M.A., Carey C.C., Hamilton D.P., Huisman J., Paerl H.W., Wood S., Wulff A. (2020). Perspective: Advancing the research agenda for improving understanding of cyanobacteria in a future of global change. Harmful Algae.

[11] Dai R., Li Z., Yan F., An L., Du W., Li X. (2023). Evaluation of changes in *M. aeruginosa* growth and microcystin production under phosphorus starvation via transcriptomic surveys. Sci. Total Environ..

[12] Buratti F.M., Manganelli M., Vichi S., Stefanelli M., Scardala S., Testai E., Funari E. (2017). Cyanotoxins: Producing organisms, occurrence, toxicity, mechanism of action and human health toxicological risk evaluation. Arch. Toxicol..

[13] Humbert J.F., Fastner J. (2016). Ecology of cyanobacteria. Handbook of Cyanobacterial Monitoring and Cyanotoxin Analysis.

[14] Janssen A.B.G., Janse J.H., Beusen A.H.W., Chang M., Harrison J.A., Huttunen I., Kong X., Rost J., Teurlincx S., A Troost T. (2019). How to model algal blooms in any lake on earth. Curr. Opin. Environ. Sustain..

[15] Huisman J., Codd G.A., Paerl H.W., Ibelings B.W., Verspagen J.M., Visser P.M. (2018). Cyanobacterial blooms. Nat. Rev. Microbiol..

[16] Igwaran A., Kayode A.J., Moloantoa K.M., Khetsha Z.P., Unuofin J.O. (2024). Cyanobacteria harmful algae blooms: Causes, impacts, and risk management. Water Air Soil Pollut..

[17] Wood S., Kelly L., Bouma-Gregson K., Humbert J.F., Laughinghouse H.D., Lazorchak J., McAllister T., McQueen A., Pokrzywinski K., Puddick J. (2020). Toxic benthic freshwater cyanobacterial proliferations: Challenges and solutions for enhancing knowledge and improving monitoring and mitigation. Freshw. Biol..

[18] Schets F.M., Van der Oost R., Van de Waal D.B. (2020). Cyanobacteria Protocol 2020.

[19] Khiari D. (2019). Managing Cyanotoxins.

[20] Wurtsbaugh W.A., Paerl H.W., Dodds W.K. (2019). Nutrients, eutrophication and harmful algal blooms along the freshwater to marine continuum. Wiley Interdiscip. Rev. Water.

[21] Reinl K.L., Harris T.D., Elfferich I., Coker A., Zhan Q., Domis L.N.D.S., Morales-Williams A.M., Bhattacharya R., Grossart H.P., North R.L. (2022). The role of organic nutrients in structuring freshwater phytoplankton communities in a rapidly changing world. Water Res..

[22] Lyche Solheim A., Gundersen H., Mischke U., Skjelbred B., Nejstgaard J.C., Guislain A.L., Sperfeld E., Giling D.P., Haande S., Ballot A. (2024). Lake browning counteracts cyanobacteria responses to nutrients: Evidence from phytoplankton dynamics in large enclosure experiments and comprehensive observational data. Glob. Change Biol..

[23] Paerl H.W., Scott J.T., McCarthy M.J., Newell S.E., Gardner W.S., Havens K.E., Hoffman D.K., Wilhelm S.W., Wurtsbaugh W.A. (2016). It takes two to tango: When and where dual nutrient (N & P) reductions are needed to protect lakes and downstream ecosystems. Environ. Sci. Technol..

[24] Deng D., Meng H., Ma Y., Guo Y., Wang Z., He H., Xie W., Liu J.E., Zhang L. (2024). The cumulative impact of temperature and nitrogen availability on the potential nitrogen fixation and extracellular polymeric substances secretion by Dolichospermum. Harmful Algae.

[25] Du X., Liu H., Yuan L., Wang Y., Ma Y., Wang R., Chen X., Losiewicz M.D., Guo H., Zhang H. (2019). The Diversity of Cyanobacterial Toxins on Structural Characterization, Distribution and Identification: A Systematic Review. Toxins.

[26] Hayes N.M., Haig H.A., Simpson G.L., Leavitt P.R. (2020). Effects of lake warming on the seasonal risk of toxic cyanobacteria exposure. Limnol. Oceanogr. Lett..

[27] Turner A.D., Hatfield R.G., Maskrey B.H., Algoet M. (2019). Evaluation of the new European Union reference method for paralytic shellfish toxins in shellfish: A review of twelve years regulatory monitoring using pre-column oxidation LC-FLD. Trends Anal. Chem..

[28] Yang Z., Zhang M., Yu Y., Shi X. (2020). Temperature triggers the annual cycle of Microcystis, comparable results from the laboratory and a large shallow lake. Chemosphere.

[29] Rigosi A., Carey C.C., Ibelings B.W., Brookes J.D. (2014). The interaction between climate warming and eutrophication to promote cyanobacteria is dependent on trophic state and varies among taxa. Limnol. Oceanogr..

[30] Ibelings B.W., Backer L.C., Kardinaal E.A., Chorus I. (2015). Current approaches to cyanotoxin risk assessment and risk management around the globe. Harmful Algae.

[31] Campos A., Redouane E.M., Freitas M., Amaral S., Azevedo T., Loss L., Máthé C., Mohamed Z.A., Oudra B., Vasconcelos V. (2021). Impacts of microcystins on morphological and physiological parameters of agricultural plants: A review. Plants.

[32] Di Pofi G. (2020). High Resolution Mass Spectrometry: New Methods of Analysis for Risk Assessment by Cyanotoxins and Cyromazine in Water for Human Consumption. Ph.D. Thesis.

[33] Hilborn E.D., Beasley V.R. (2015). One health and cyanobacteria in freshwater systems: Animal illnesses and deaths are sentinel events for human health risks. Toxins.

[34] Hitzfeld B.C., Höger S.J., Dietrich D.R. (2000). Cyanobacterial toxins: Removal during drinking water treatment, and human risk assessment. Environ. Health Perspect..

[35] Caen A., Mathias J.D., Latour D. (2024). How do seasonal temperature variations influence interplay between toxic and non-toxic cyanobacterial blooms? Evidence from modeling and experimental data. Harmful Algae.

[36] Falfushynska H., Kasianchuk N., Siemens E., Henao E., Rzymski P.A. (2023). Review of Commonv Cyanotoxins and Their Effects on Fish. Toxics.

[37] Leavitt P.R., Findlay D.L. (1994). Comparison of fossil pigments with 20 years of phytoplankton data from eutrophic Lake 227, Experimental Lakes area, Ontario. Can. J. Fish. Aquat. Sci..

[38] Waters M.N., Brenner M., Curtis J.H., Romero-Oliva C.S., Dix M., Cano M. (2021). Harmful algal blooms and cyanotoxins in Lake Amatitlán, Guatemala, coincided with ancient Maya occupation in the watershed. Proc. Natl. Acad. Sci. USA.

[39] Heathcote A.J., Taranu Z.E., Tromas N., MacIntyre-Newell M., Leavitt P.R., Pick F.R. (2024). Sedimentary DNA and pigments show increasing abundance and toxicity of cyanoHABs during the Anthropocene. Freshw. Biol..

[40] Dick G.J., Duhaime M.B., Evans J.T., Errera R.M., Godwin C.M., Kharbush J.J., Nitschky H.S., Powers M.A., Vanderploeg H.A., Schmidt K.C. (2021). The genetic and ecophysiological diversity of Microcystis. Environ. Microbiol..

[41] Xiao M., Li M., Reynolds C.S. (2018). Colony formation in the cyanobacterium Microcystis. Biol. Rev..

[42] Poulickova A., Lysakova M., Hasler P., Lelkova E. (2008). Fishpond sediments—The source of palaeoecological information and algal “seed banks”. Nova Hedwig..

[43] Kaplan-Levy R.N., Hadas O., Summers M.L., Rücker J., Sukenik A. (2010). Akinetes: Dormant cells of cyanobacteria. Dormancy and Resistance in Harsh Environments.

[44] Kravchuk E.S., Ivanova E.A., Gladyshev M.I. (2006). Seasonal dynamics of akinetes of Anabaena flos-aquae in bottom sediments and water column of small Siberian reservoir. Aquat. Ecol..

[45] Salazar Torres G., Adámek Z. (2013). Factors promoting the recruitment of benthic cyanobacteria resting stages: A review. Croat. J. Fish. Ribar..

[46] Gyllstrom M., Hansson L.A. (2004). Dormancy in freshwater zooplankton: Induction, termination and the importance of benthicpelagic coupling. Aquat. Sci..

[47] Suikkanen S., Kaartokallio H., Hällfors S., Huttunen M., Laamanen M. (2010). Life cycle strategies of bloom-forming, filamentous cyanobacteria in the Baltic Sea. Deep Sea Res. Part II Top. Stud. Oceanogr..

[48] Cottingham K.L., Weathers K.C., Ewing H.A., Greer M.L., Carey C.C. (2021). Predicting the effects of climate change on freshwater cyanobacterial blooms requires consideration of the complete cyanobacterial life cycle. J. Plankton Res..

[49] Amzil Z., Derrien A., Terre Terrillon A., Savar V., Bertin T., Peyrat M., Duval A., Lhaute K., Arnich N., Hort V. (2023). Five years monitoring the emergence of unregulated toxins in shellfish in France (EMERGTOX 2018–2022). Mar. Drugs.

[50] Carmichael W.W., Boyer G.L. (2016). Health impacts from cyanobacteria harmful algae blooms: Implications for the North American Great Lakes. Harmful Algae.

[51] Smith R.B., Bass B., Sawyer D., Depew D., Watson S.B. (2019). Estimating the economic costs of algal blooms in the Canadian Lake Erie Basin. Harmful Algae.

[52] Chorus I., Welker M. (2021). Toxic Cyanobacteria in Water: A Guide to Their Public Health Consequences, Monitoring and Management.

[53] WHO (2021). Guidelines on Recreational Water Quality. Volume 1: Coastal and Fresh Waters.

[54] Holland A., Kinnear S. (2013). Interpreting the possible ecological role (s) of cyanotoxins: Compounds for competitive advantage and/or physiological aide?. Mar. Drugs.

[55] Jeong J.Y., Lee S.H., Yun M.R., Oh S.E., Hwang C.W., Yoon M.H., Park H.D. (2020). Draft genome sequence of Raphidiopsis raciborskii strain GIHE 2018, isolated from a shallow freshwater pond in South Korea. Microbiol. Resour. Announc..

[56] Christensen V.G., Khan E. (2020). Freshwater neurotoxins and concerns for human, animal, and ecosystem health: A review of anatoxin-a and saxitoxin. Sci. Total Environ..

[57] Mantzouki E., Lürling M., Fastner J., de Senerpont Domis L., Wilk-Woniak E., Koreivien J., Warming T.P. (2018). Temperature effects explain continental scale distribution of cyanobacterial toxins. Toxins.

[58] Hinojosa M.G., Gutiérrez-Praena D., Prieto A.I., Guzmán-Guillén R., Jos A., Cameán A.M. (2019). Neurotoxicity induced by microcystins and cylindrospermopsin: A review. Sci. Total Environ..

[59] Van Apeldoorn M.E., Van Egmond H.P., Speijers G.J., Bakker G.J. (2007). Toxins of cyanobacteria. Mol. Nutr. Food Res..

[60] Sivonen K., Jones G. (1999). Cyanobacterial toxins. Toxic Cyanobacteria in Water: A Guide to Their Public Health Consequences, Monitoring and Management.

[61] Funari E., Testai E. (2008). Human health risk assessment related to cyanotoxins exposure. Crit. Rev. Toxicol..

[62] Jones G.J., Orr P.T. (1994). Release and degradation of microcystin following algicide treatment of a *Microcystis aeruginosa* bloom in a recreational lake, as determined by HPLC and protein phosphatase inhibition assay. Water Res..

[63] Chorus I., Bartram J. (1999). Toxic Cyanobacteria in Water—A Guide to Their Public Health Consequences, Monitoring and Management.

[64] Roy-Lachapelle A., Solliec M., Sauvé S., Gagnon C. (2021). Evaluation of ELISA-based method for total anabaenopeptins determination and comparative analysis with on-line SPE-UHPLC-HRMS in freshwater cyanobacterial blooms. Talanta.

[65] Chen Y., Shen D., Fang D. (2013). Nodularins in poisoning. Clin. Chim. Acta.

[66] Carmichael W.W., Azevedo S.M., Jochimsen E.M., Lau S., Rinehart K.L., Shaw G.R., Eaglesham G.K. (2001). Human fatalities from cyanobacteria: Chemical and biological evidence for cyanotoxins. Environ. Health Perspect..

[67] Kalaitzidou M., Petridou E., Economou V., Theodoridis A., Angelidis P. (2016). Toxic cyanobacteria. A biological hazard for animal and public health: A review. Air Water Borne Dis..

[68] Thajuddin F., Rasheed A.S., Thajuddin N., Dhanasekaran D. (2023). Identification of Microcystin, Nodularin Synthatase Gene Clusters in Toxic Cyanobacteria Using AntiSMASH Pipeline. Protocols for Cyanobacteria Sampling and Detection of Cyanotoxin.

[69] Abdallah M.F., Van Hassel W.H., Andjelkovic M., Wilmotte A., Rajkovic A. (2021). Cyanotoxins and food contamination in developing countries: Review of their types, toxicity, analysis, occurrence and mitigation strategies. Toxins.

[70] Díez-Quijada L., Puerto M., Gutiérrez-Praena D., Llana-Ruiz-Cabello M., Jos A., Cameán A.M. (2019). Microcystin-RR: Occurrence, content in water and food and toxicological studies. A review. Environ. Res..

[71] Kalaitzidou M.P., Nannou C.I., Lambropoulou D.A., Papageorgiou K.V., Theodoridis A.M., Economou V.K., Giantsis I.A., Angelidis P.G., Kritas S.K., Petridou E.J. (2021). First report of detection of microcystins in farmed mediterranean mussels *Mytilus galloprovincialis* in Thermaikos gulf in Greece. J. Biol. Res..

[72] Carmichael W.W., Eschedor J.T., Patterson G.M., Moore R.E. (1988). Toxicity and partial structure of a hepatotoxic peptide produced by the cyanobacterium *Nodularia spumigena* Mertens emend. Appl. Environ. Microbiol..

[73] Falconer I.R., Humpage A.R. (2005). Health risk assessment of cyanobacterial (blue-green algal) toxins in drinking water. Int. J. Environ. Res. Public Health.

[74] Miller T.R., Beversdorf L.J., Weirich C.A., Bartlett S.L. (2017). Cyanobacterial Toxins of the Laurentian Great Lakes, Their Toxicological Effects, and Numerical Limits in Drinking Water. Mar. Drugs.

[75] Jones M.R., Pinto E., Torres M.A., Dörr F., Mazur-Marzec H., Szubert K., Tartaglione L., Dell’Aversano C., Miles C.O., Beach D.G. (2021). CyanoMetDB, a comprehensive public database of secondary metabolites from cyanobacteria. Water Res..

[76] He X., Liu Y.L., Conklin A., Westrick J., Weavers L.K., Dionysiou D.D., Lenhart J.J., Mouser P.J., Szlag D., Walker H.W. (2016). Toxic cyanobacteria and drinking water: Impacts, detection, and treatment. Harmful Algae.

[77] Krishnamurthy T., Carmichael W.W., Sarver E.W. (1986). Toxic peptides from freshwater cyanobacteria (blue-green algae). I. Isolation, purification and characterization of peptides from *Microcystis aeruginosa* and *Anabaena flos-aquae*. Toxicon.

[78] Watanabe M.F., Oishi S., Harada K.I., Matsuura K., Kawai H., Suzuki M. (1988). Toxins contained in *Microcystis* species of cyanobacteria (blue-green algae). Toxicon.

[79] Lyon-Colbert A., Su S., Cude C. (2018). A Systematic Literature Review for Evidence of *Aphanizomenon flos-aquae* Toxigenicity in Recreational Waters and Toxicity of Dietary Supplements: 2000–2017. Toxins.

[80] Rodrigues M.A., Reis M.P., Mateus M.C. (2013). Liquid chromatography/negative electrospray ionization ion trap MS2 mass spectrometry application for the determination of microcystins occurrence in Southern Portugal water reservoirs. Toxicon.

[81] Vasconcelos V. (2010). Molecular mechanisms of microcystin toxicity in animal cells. Int. J. Mol. Sci..

[82] Fontanillo M., Köhn M. (2018). Microcystins: Synthesis and structure-activity relationship studies toward PP1 and PP2A. Bioorg. Med. Chem..

[83] Swingle M.R., Honkanen R.E. (2019). Inhibitors of serine/threonine protein phosphatases: Biochemical and structural studies provide insight for further development. Curr. Med. Chem..

[84] MacKintosh C., Beattie K.A., Klumpp S., Cohen P., Codd G.A. (1990). Cyanobacterial microcystin-LR is a potent and specific inhibitor of protein phosphatases 1 and 2A from both mammals and higher plants. FEBS Lett..

[85] Runnegar M., Berndt N., Kong S.M., Lee E.Y.C., Zhang L.F. (1995). In-vivo and in-vitro binding of microcystin to protein phosphatase-1 and phosphatase-2a. Biochem. Biophys. Res. Commun..

[86] Carmichael W.W. (1992). Cyanobacteria secondary metabolites-the cyanotoxins. J. Appl. Bacteriol..

[87] Arman T., Clarke J.D. (2021). Microcystin toxicokinetics, molecular toxicology, and pathophysiology in preclinical rodent models and humans. Toxins.

[88] Jos A., Pichardo S., Prieto A.I., Repetto G., Vázquez C.M., Moreno I., Cameán A.M. (2005). Toxic cyanobacterial cells containing microcystins induce oxidative stress in exposed tilapia fish (*Oreochromis* sp.) under laboratory conditions. Aquat. Toxicol..

[89] Zhang J., Wang N., Zhang Z., Gao Y., Dong J., Gao X., Yuan H., Li X. (2023). The Combined Effects of Toxic *Microcystis aeruginosa* and Thermal Stress on the Edible Clam *(Corbicula fluminea*): Insights into Oxidative Stress Responses and Molecular Networks. Antioxidants.

[90] WHO (2020). Cyanobacterial toxins: Microcystins. Background Document for Development of WHO Guidelines for Drinking-Water Quality and Guidelines for Safe Recreational Water Environments.

[91] Melaram R., Newton A.R., Lee A., Herber S., El-Khouri A., Chafin J. (2024). A review of microcystin and nodularin toxins derived from freshwater cyanobacterial harmful algal blooms and their impact on human health. Toxicol. Environ. Health Sci..

[92] Zhou L., Yu D., Yu H., Chen K., Shen G., Shen Y., Ruan Y., Ding X. (2000). Drinking water types, microcystins and colorectal cancer. Zhonghua Yu Fang Yi Xue Za Zhi Chin. J. Prev. Med..

[93] IARC (2024). Monographs on the Identification of Carcinogenic Hazards to Humans.

[94] Yoshida T., Makita Y., Nagata S., Tsutsumi T., Yoshida F., Sekijima M., Tamura S., Ueno Y. (1997). Acute oral toxicity of microcystin-LR, a cyanobacterial hepatotoxin, in mice. Nat. Toxins.

[95] Ostry V., Malir F., Toman J., Grosse Y. (2017). Mycotoxins as human carcinogens-the IARC monographs classification. Mycotoxin Res..

[96] Nehring S. (1993). Mechanisms for Recurrent Nuisance Algal Blooms in Coastal Zones: Resting Cyst Formation as Life-Strategy of Dinoflagellates.

[97] Farrer D., Counter M., Hillwig R., Cude C. (2015). Health-Based Cyanotoxin Guideline Values Allow for Cyanotoxin-Based Monitoring and Efficient Public Health Response to Cyanobacterial Blooms. Toxins.

[98] Humpage A.R., Falconer I.R. (2003). Oral toxicity of the cyanobacterial toxin cylindrospermopsin in male Swiss albino mice: Determination of no observed adverse effect level for deriving a drinking water guideline value. Environ. Toxicol..

[99] Stevens D.K., Krieger R.I. (1991). Stability studies on the cyanobacterial nicotinic alkaloid anatoxin-a. Toxicon.

[100] Cook W.O., Dellinger J.A., Singh S.S., Dahlem A.M., Carmichael W.W., Beasley V.R. (1989). Regional brain cholinesterase activity in rats injected intraperitoneally with anatoxin-a(s) or paraoxon. Toxicol. Lett..

[101] Testai E., Buratti F.M., Funari E., Manganelli M., Vichi S., Arnich N., Biré R., Fessard V., Sialehaamoa A. (2016). Review and analysis of occurrence, exposure and toxicity of cyanobacteria toxins in food. EFSA Support. Publ..

[102] Melo E., Moura e Silva S., Paula F.R. (2014). Molecular modelling and quantitative structure-activity relationship studies of anatoxin-a and epibatidine derivatives with affinity to rodent nAChR receptors. Chem. Pap..

[103] European Food Safety Authority (EFSA) (2009). Marine biotoxins in shellfish-Saxitoxin group. EFSA J..

[104] Simiyu B.M., Oduor S.O., Rohrlack T., Sitoki L., Kurmayer R. (2018). Microcystin Content in Phytoplankton and in Small Fish from Eutrophic Nyanza Gulf, Lake Victoria, Kenya. Toxins.

[105] Calado S.L.D.M., Santos G.S., Leite T.P.B., Wojciechowski J., Nadaline M., Bozza D.C., Freitas de Magalhães V., Cestari M.M., Prodocimo V., Silva de Assis H.C. (2018). Depuration time and sublethal effects of microcystins in a freshwater fish from water supply reservoir. Chemosphere.

[106] Miller M.A., Kudela R.M., Mekebri A., Crane D., Oates S.C., Tinker M.T., Staedler M., Miller A., Toy-Choutka S., Dominik C. (2010). Evidence for a Novel Marine Harmful Algal Bloom: Cyanotoxin (Microcystin) Transfer from Land to Sea Otters. PLoS ONE.

[107] Van der Merwe D., Sebbag L., Nietfeld J.C., Aubel M.T., Foss A., Carney E. (2012). Investigation of a *Microcystis aeruginosa* cyanobacterial freshwater harmful algal bloom associated with acute microcystin toxicosis in a dog. J. Vet. Diagn. Investig..

[108] Singo A., Myburgh J.G., Laver P.N., Venter E.A., Ferreira G.C.H., Rösemann G.M., Botha C.J. (2017). Vertical transmission of microcystins to Nile crocodile (*Crocodylus niloticus*) eggs. Toxicon.

[109] Malbrouck C., Kestemont P. (2006). Effects of microcystins on fish. Environ. Toxicol. Chem. Int. J..

[110] Pitois F., Vezie C., Thoraval I., Baurès E. (2016). Improving microcystin monitoring relevance in recreative waters: A regional case-study (Brittany, Western France, Europe). Int. J. Hyg. Environ. Health.

[111] Schaefer A.M., Yrastorza L., Stockley N., Harvey K., Harris N., Grady R., Sullivan J., McFarland M., Reif J.S. (2020). Exposure to microcystin among coastal residents during a cyanobacteria bloom in Florida. Harmful Algae.

[112] Fischer W.J., Altheimer S., Cattori V., Meier P.J., Dietrich D.R., Hagenbuch B. (2005). Organic anion transporting polypeptides expressed in liver and brain mediate uptake of microcystin. Toxicol. Appl. Pharmacol..

[113] Chen L., Chen J., Zhang X., Xie P. (2015). A review of reproductive toxicity of microcystins. J. Hazard. Mater..

[114] Lu Y., Zhang X., Zheng P., Li J., Li J., Li T., Wang X., Wang D., Xian J., Zhang Z. (2023). Effects of microcystin-LR on behavior, histopathology, oxidative stress, non-specific immunity and gene expression of red claw crayfish (*Cherax quadricarinatus*). Aquacult. Rep..

[115] Hernandez B.Y., Zhu X., Risch H.A., Lu L., Ma X., Irwin M.L., Lim J.K., Taddei T.H., Pawlish K.S., Stroup A.M. (2022). Oral Cyanobacteria and Hepatocellular Carcinoma. Cancer Epidemiol. Biomark. Prev..

[116] Christophoridis C., Zervou S.K., Manolidi K., Katsiapi M., Moustaka-Gouni M., Kaloudis T., Triantis T.M., Hiskia A. (2018). Occurrence and diversity of cyanotoxins in Greek lakes. Sci. Rep..

[117] Foss A.J., Butt J., Fuller S., Cieslik K., Aubel M.T., Wertz T. (2017). Nodularin from benthic freshwater periphyton and implications for trophic transfer. Toxicon.

[118] Sivonen K., Kononen K., Carmichael W.W., Dalem A.M., Rinehart K.L., Kiviranta J., Niemela S.I. (1989). Occurrence of the hepatotoxic cyanobacterium *Nodularia spumigena* in the Baltic Sea and structure of the toxin. Appl. Environ. Microbiol..

[119] Bownik A. (2016). Harmful algae: Effects of cyanobacterial cyclic peptides on aquatic invertebrates-a short review. Toxicon.

[120] Konkel R., Toruska-Sitarz A., Cegowska M., Ežerinskis Ž., Šapolaitė J., Mažeika J., Mazur-Marzec H. (2020). Blooms of toxic cyanobacterium *Nodularia spumigena* in *Norwegian fjords* during Holocene warm periods. Toxins.

[121] Kaur H.P., Prasad B., Kaur S. (2015). A review on applications of biosurfactants produced from unconventional inexpensive wastes in food and agriculture industry. World J. Pharm. Res..

[122] Lopes V.R., Vasconcelos V.M. (2011). Planktonic and benthic cyanobacteria of European brackish waters: A perspective on estuaries and brackish seas. Eur. J. Phycol..

[123] Ufelmann H., Krüger T., Luckas B., Schrenk D. (2012). Human and rat hepatocyte toxicity and protein phosphatase 1 and 2A inhibitory activity of naturally occurring desmethyl-microcystins and nodularins. Toxicology.

[124] Bagu J.R., Sykes B.D., Craig M.M., Holmes C.B. (1997). A molecular basis for different interactions of marine toxins with protein phosphatase-1: Molecular models for bound motuporin, microcystins, okadaic acid, and calyculin A. J. Biol. Chem..

[125] Teikari J.E., Hou S., Wahlsten M., Hess W.R., Sivonen K. (2018). Comparative Genomics of the Baltic Sea Toxic Cyanobacteria *Nodularia spumigena* UHCC 0039 and Its Response to Varying Salinity. Front. Microbiol..

[126] Liu P., Lu Z., Liu L., Li R., Liang Z., Shen M., Xu H., Ren D., Ji M., Yuan S. (2019). NOD-like receptor signaling in inflammation-associated cancers: From functions to targeted therapie. Phytomedicine.

[127] Carmichael W.W. (2001). Health effects of toxin producing cyanobacteria: “The CyanoHABS”. Hum. Ecol. Risk Assess..

[128] Stucken K., John U., Soto-Liebe K., Vásquez M. (2014). Impact of nitrogen sources on gene expression and toxin production in the diazotroph *Cylindrospermopsis raciborskii* CS-505 and non-diazotroph *Raphidiopsis brookii* D9. Toxins.

[129] Jiang Y., Song G., Pan Q., Yang Y., Li R. (2015). Identification of genes for Anatoxin-a biosynthesis in *Cuspidothrix issatschenkoi*. Harmful Algae.

[130] Loftin K.A., Graham J.L., Hilborn E., Lehmann S., Meyer M.T., Dietze J.E., Griffith C. (2016). Cyanotoxins in inland lakes of the United States: Occurrence and potential recreational health risks in the EPA National Lakes Assessment 2007. Harmful Algae.

[131] Salmaso N., Cerasino L., Boscaini A., Capelli C. (2016). Planktic *Tychonema* (Cyanobacteria) in the large lakes south of the Alps: Phylogenetic assessment and toxigenic potential. FEMS Microbiol. Ecol..

[132] Smith C., Sutton A. (1993). The Persistence of Anatoxin-A in Reservoir Water.

[133] WHO (2020). Cyanobacterial toxins: Anatoxin-a and analogues. Background Document for Development of WHO Guidelines for Drinking-Water Quality and Guidelines for Safe Recreational Water Environments.

[134] Al-Sammak M.A., Hoagland K.D., Cassada D., Snow D.D. (2014). Co-occurrence of the cyanotoxins BMAA, DABA and anatoxin-a in Nebraska reservoirs, fish, and aquatic plants. Toxins.

[135] Jaramillo M., O’Shea K.E. (2019). Analytical methods for assessment of cyanotoxin contamination in drinking water sources. Curr. Opin. Environ. Sci. Health.

[136] Plata-Calzado C., Prieto A.I., Cameán A.M., Jos A. (2024). Analytical Methods for Anatoxin-a Determination: A Review. Toxins.

[137] Patoka J., Gupta R.C., Kua K. (2011). Anatoxin-a(s): Natural organophosphorus anticholinesterase agent. Mil. Med. Sci. Lett..

[138] Ferrão-Filho A.D.S., Kozlowsky-Suzuki B. (2011). Cyanotoxins: Bioaccumulation and effects on aquatic animals. Mar. Drugs.

[139] Matsunaga S., Moore R.E., Niemczura W.P., Carmichael W.W. (1989). Anatoxin-a (s), a potent anticholinesterase from Anabaena flos-aquae. J. Am. Chem. Soc..

[140] McAllister T.G., Wood S., Hawes I. (2016). The rise of toxic benthic Phormidium proliferations: A review of their taxonomy, distribution, toxin content and factors regulating prevalence and increased severity. Harmful Algae.

[141] Colas S., Marie B., Lance E., Quiblier C., Tricoire-Leignel H., Mattei C. (2021). Anatoxin-a: Overview on a harmful cyanobacterial neurotoxin from the environmental scale to the molecular target. Environ. Res..

[142] Botana L.M., Hess P., Munday R., Nathalie A., DeGrasse S.L., Feeley M., Suzuki T., van den Berg M., Fattori V., Garrido Gamarro E. (2017). Derivation of toxicity equivalency factors for marine biotoxins associated with Bivalve Molluscs. Trends Food Sci. Technol..

[143] Turner A.D., Dhanji-Rapkova M., O’Neill A., Coates L., Lewis A., Lewis K. (2018). Analysis of Microcystins in Cyanobacterial Blooms from Freshwater Bodies in England. Toxins.

[144] Oyaneder-Terrazas J., Contreras H.R., García C. (2017). Prevalence, Variability and Bioconcentration of Saxitoxin-Group in Different Marine Species Present in the Food Chain. Toxins.

[145] Andrinolo D., Iglesias V., García C., Lagos N. (2002). Toxicokinetics and toxicodynamics of gonyautoxins after an oral toxin dose in cats. Toxicon.

[146] Zhang D.W., Deng X., Xie P., Chen J., Guo L. (2013). Risk assessment of microcystins in silver carp (*Hypophthalmichthys molitrix*) from eight eutrophic lakes in China. Food Chem..

[147] Fuentes-Valdés J.J., Soto-Liebe K., Pérez-Pantoja D., Tamames J., Belmar L., Pedrós-Alió C., Garrido D., Vásquez M. (2018). Draft genome sequences of *Cylindrospermopsis raciborskii* strains CS-508 and MVCC14, isolated from freshwater bloom events in Australia and Uruguay. Stand. Genom. Sci..

[148] Fedorova G.A., Kuzmin A.V., Zubkov I.N., Tikhonova I.V., Shtykova Y.R., Butina T.V., Belykh O.I., Grachev M.A. (2019). Determination of saxitoxin in water of Lake Baikal. Acta Biol. Sib..

[149] Jones G.J., Negri A.P. (1997). Persistence and degradation of cyanobacterial paralytic shellfish poisons (PSPs) in freshwaters. Water Res..

[150] Pereira P., Onodera H., Andrinolo D., Franca S., Araújo F., Lagos N., Oshima Y. (2000). Paralytic shellfish toxins in the freshwater cyanobacterium *Aphanizomenon flos-aquae*, isolated from Montargil reservoir, Portugal. Toxicon.

[151] Figueiredo D.R., Azeiteiro U.M., Esteves S.M., Gonçalves F.J.M., Pereira M.J. (2004). Microcystin-producing blooms-a serious global public health issue. Ecotoxicol. Environ. Saf..

[152] Harland F., Wood S.A., Broady P., Williamson W., Gaw S. (2015). Changes in saxitoxin production through growth phases in the metaphytic cyanobacterium *Scytonema* cf. *crispum*. Toxicon.

[153] Batoréu M.C., Dias E., Pereira P., Franca S. (2005). Risk of human exposure to paralytic toxins of algal origin. Environ. Toxicol. Pharmacol..

[154] Trainer V.L., Hardy F.J. (2015). Integrative monitoring of marine and freshwater harmful algae in Washington State for public health protection. Toxins.

[155] Aguilera A., Giannuzzi L. (2018). Book Review: Cyanobacteria as Environmental Determinants of Health.

[156] Garcia M.R., Mirlean N., Baisch P.R., Caramao E.B. (2010). Assessment of polycyclic aromatic hydrocarbon influx and sediment contamination in an urbanized estuary. Environ. Monit. Assess..

[157] García C., Bravo M.C., Lagos M., Lagos N. (2004). Paralytic shellfish poisoning: Post-mortem analysis of tissue and body fluid samples from human victims in the Patagonia fjords. Toxicon.

[158] García C., Rodriguez-Navarro A., Díaz J.C., Torres R., Lagos N. (2009). Evidence of in vitro glucuronidation and enzymatic transformation of paralytic shellfish toxins by healthy human liver microsomes fraction. Toxicon.

[159] Garcia C., Barriga A., Diaz J.C., Lagos M., Lagos N. (2010). Route of metabolization and detoxication of paralytic shellfish toxins in humans. Toxicon.

[160] Masias D., Gómez K., Contreras C., Gaete L., Garcia C. (2019). Rapid Screening Fluorescence method applied to detection and quantitation of Paralytic Shellfish Toxins in invertebrate marine vectors. Food Addit. Contam. Part A.

[161] EU (2017). Commission regulation (EC) No 2017/1980 of 31 October 2017 amending Annex III to Regulation (EC) No 2074/2005 as regards paralytic shellfish poison (PSP) detection method. Off. J. Eur. Union.

[162] WHO (2020). Cyanobacterial toxins: Saxitoxins. Background Document for Development of WHO Guidelines for Drinking-Water Quality and Guidelines for Safe Recreational Water Environments.

[163] Berdalet E., Fleming L.E., Gowen R., Davidson K., Hess P., Backer L.C., Moore S.K., Hoagland P., Enevoldsen H. (2015). Marine harmful algal blooms, human health and wellbeing: Challenges and opportunities in the 21st century. J. Mar. Biol. Assoc. UK.

[164] Rzymski P., Poniedziałek B. (2014). In search of environmental role of cylindrospermopsin: A review on global distribution and ecology of its producers. Water Res..

[165] Ohtani I., Moore R.E., Runnegar M.T.C. (1991). Cylindrospermopsin: A potent hepatotoxin from the blue-green alga *Cylindrospermopsis raciborskii*. J. Am. Chem. Soc..

[166] WHO (2020). Cyanobacterial toxins: Cylindrospermopsins. Background Document for Development of WHO Guidelines for Drinking-Water Quality and Guidelines for Safe Recreational Water Environments.

[167] Sant Anna C., de Carvalho L.R., Fiore M.F., Silva-Stenico M.E., Lorenzi A.S., Rios F.R., Konno K., Garcia C., Lagoa N. (2011). Highly toxic *Microcystis aeruginosa* strain, isolated from Sao Paulo-Brazil, produce Hepatotoxins and Paralytic Shellfish Toxins. Neurotox. Res..

[168] Davis D.A., Mondo K., Stern E., Annor A.K., Murch S.J., Coyne T.M., Brand L.E., Niemeyer M.E., Sharp S., Bradley W.G. (2019). Cyanobacterial neurotoxin BMAA and brain pathology in stranded dolphins. PLoS ONE.

[169] Rücker J., Stüken A., Nixdorf B., Fastner J., Chorus I., Wiedner C. (2007). Concentrations of particulate and dissolved cylindrospermopsin in 21 Aphanizomenon-dominated temperate lakes. Toxicon.

[170] Chiswell R.K., Shaw G.R., Eaglesham G., Smith M.J., Norris R.L., Seawright A.A., Moore M.R. (1999). Stability of cylindrospermopsin, the toxin from the cyanobacterium, *Cylindrospermopsis raciborskii*: Effect of pH, temperature, and sunlight on decomposition. Environ. Toxicol. Int. J..

[171] Wormer L., Cirés S., Carrasco D., Quesada A. (2008). Cylindrospermopsin is not degraded by co-occurring natural bacterial communities during a 40-day study. Harmful Algae.

[172] Sukenik A., Kaplan A. (2021). Cyanobacterial harmful algal blooms in aquatic ecosystems: A comprehensive outlook on current and emerging mitigation and control approaches. Microorganisms.

[173] Terao K., Ohmori S., Igarashi K., Ohtani I., Watanabe M.F., Harada K.I., Ito E., Watanabe M. (1994). Electron microscopic studies on experimental poisoning in mice induced by cylindrospermopsin isolated from blue-green alga *Umezakia natans*. Toxicon.

[174] Froscio S.M., Humpage A.R., Burcham P.C., Falconer I.R. (2003). Cylindrospermopsin-induced protein synthesis inhibition and its dissociation from acute toxicity in mouse hepatocytes. Environ. Toxicol. Int. J..

[175] Runnegar M.T., Xie C., Snider B.B., Wallace G.A., Weinreb S.M., Kuhlenkamp J. (2002). In vitro hepatotoxicity of the cyanobacterial alkaloid cylindrospermopsin and related synthetic analogues. Toxicol. Sci..

[176] Wiegand C., Pflugmacher S. (2005). Ecotoxicological effects of selected cyanobacterial secondary metabolites a short review. Toxicol. Appl. Pharmacol..

[177] Chong M.W.K., Wong B.S.F., Lam P.K.S., Shaw G.R., Seawright A.A. (2002). Toxicity and uptake mechanism of cylindrospermopsin and lophyrotomin in primary rat hepatocytes. Toxicon.

[178] Yang Y., Yu G., Chen Y., Jia N., Li R. (2021). Four decades of progress in cylindrospermopsin research: The ins and outs of a potent cyanotoxin. J. Hazard. Mater..

[179] Štraser A., Filipič M., Žegura B. (2011). Genotoxic effects of the cyanobacterial hepatotoxin cylindrospermopsin in the HepG2 cell line. Arch. Toxicol..

[180] Puerto M., Prieto A.I., Maisanaba S., Gutiérrez-Praena D., Mellado-García P., Jos Á., Cameán A.M. (2018). Mutagenic and genotoxic potential of pure Cylindrospermopsin by a battery of in vitro tests. Food Chem. Toxicol..

[181] Pichardo S., Cameán A.M., Jos A. (2017). In vitro toxicological assessment of cylindrospermopsin: A review. Toxins.

[182] Falconer I.R., Humpage A.R. (2001). Preliminary evidence for in vivo tumour initiation by oral administration of extracts of the blue-green alga *Cylindrospermopsis raciborskii* containing the toxin cylindrospermopsin. Environ. Toxicol. Int. J..

[183] World Health Organization (2020). Cyanobacterial Toxins: Cylindrospermopsins.

[184] Huguet A., Lanceleur R., Quenault H., Le Hégarat L., Fessard V. (2019). Identification of key pathways involved in the toxic response of the cyanobacterial toxin cylindrospermopsin in human hepatic HepaRG cells. Toxicol. Vitr..

[185] Khomutovska N., Sandzewicz M., Łach Ł., Suska-Malawska M., Chmielewska M., Mazur-Marzec H., Cegłowska M., Niyatbekov T., Wood S.A., Puddick J. (2020). Limited microcystin, anatoxin and cylindrospermopsin production by cyanobacteria from microbial mats in cold deserts. Toxins.

[186] Quiblier C., Wood S., Echenique-Subiabre I., Heath M., Villeneuve A., Humbert J.F. (2013). A review of current knowledge on toxic benthic freshwater cyanobacteria—Ecology, toxin production and risk management. Water Res..

[187] Belykh O.I., Tikhonova I.V., Kuzmin A.V., Sorokovikova E.G., Fedorova G.A., Khanaev I.V., Sherbakova T.A., Timoshkin O.A. (2016). First detection of benthic cyanobacteria in Lake Baikal producing paralytic shellfish toxins. Toxicon.

[188] United States Environmental Protection Agency (2019). Cyanobacteria and Cyanotoxins: Information for Drinking Water Systems.

[189] European Union (2020). Diario Oficial de la Unión Europea. Relativa a la Calidad de las Aguas Destinadas al Consumo Humano.

[190] Australian Government (2017). Australian Drinking Water Guidelines 6 2011.

[191] Ministério de Sáude (2001). Portaria n° 1.469 Controle e Vigilância da Qualidade da Água para Consumo Humano e seu Padrão de Potabilidade.

[192] Oregon Health Autority OHA (2021). Public Health Advisory Guidelines, Harmful Algae Blooms in Freshwater Bodies.

[193] Health Canada (2022). Guidelines for Canadian Recreational Water Quality Guideline Technical Document Cyanobacteria and Their Toxins.

[194] Christoffersen K. (2005). DENMARK: Occurrence, Monitoring and Management of Toxic Cyanobacteria. Current Approaches to Cyanotoxin Risk Assessment, Risk Management and Regulations in Different Countries.

[195] Willame R., Jurczak T., Iffly J., Kull T., Meriluoto J., Hoffman L. (2005). Distribution of Hepatotoxic Cyanobacterial Blooms in Belgium and Luxembourg. Hydrobiologia.

[196] Arnich N. (2012). Regulation, Risk Management, Risk Assessment and Research on Cyanobacteria and Cyanotoxins.

[197] Rapala J., Kilponen J., Järvinen M., Lahti K. (2012). Guidelines for Monitoring of Cyanobacteria and Their Toxins.

[198] Chorus I. (2012). Approaches to assessing and managing the cyanotoxin risk. Current Approaches to Cyanotoxin Risk Assessment, Risk Management and Regulations in Different Countries.

[199] Kagalou I., Kormas K., Papadimitriou T., Katsiapi M., Genitsaris S., Moustaka-GounI M. (2012). Occurrence, Monitoring and Risk Management of Cyanobacteria and Cyanotoxins. Current Approaches to Cyanotoxin Risk Assessment, Risk Management and Regulations in Different Countries.

[200] Funari E., Gramaccioni L., Scardala S. (2012). Management of Potenially Toxic Cyanobacteria. Current Approaches to Cyanotoxin Risk Assessment, Risk Management and Regulations in Different Countries.

[201] Mankiewicz-Boczek J., Kobos J., Gągała I., Izydorczyk K. (2012). Management and Regulation of Toxic Cyanobacteria. Current Approaches to Cyanotoxin Risk Assessment, Risk Management and Regulations in Different Countries.

[202] Maršálek B., Bláha L., Bláhová L., Babica P. (2005). Management and Regulation of Cyanobacteria and Cyanotoxins. Federal Environmental Agency (Umweltbundesamt), Current Approaches to Cyanotoxin Risk Assessment, Risk Management and Regulations in Different Countries.

[203] Ministério do Ambiente, do Ordenamento do Território e do Desenvolvimento Regional (2007). Decreto-Lei, Nº 306.

[204] Schürmann Q.J., Visser P.M., Sollie S., Kardinaal W.E.A., Faassen E.J., Lokmani R., van der Oost R., Van de Waal D.B. (2024). Risk assessment of toxic cyanobacterial blooms in recreational waters: A comparative study of monitoring methods. Harmful Algae.

[205] Wood S., Williamson W. (2012). New Zealand: Regulation and Management of Cyanobacteria. Current Approaches to Cyanotoxin Risk Assessment, Risk Management and Regulations in Different Countries.

[206] Harn Te S., Yew-Hoong Gin K. (2011). The dynamics of cyanobacteria and microcystin production in a tropical reservoir of Singapore. Harmful Algae.

[207] Quesada A., de Hoyos C., Martínez G. (2012). Cyanobacteria and Cyanotoxins—Legislation and Current Situation. Current Approaches to Cyanotoxin Risk Assessment, Risk Management and Regulations in Different Countries.

[208] Koker L., Akcaalan R., Oguz A., Gaygusuz O., Gurevin C., AkatKose C., Gucver S., Karaaslan Y., Erturk A., Albay M. (2017). Distribution of toxic cyanobacteria and cyanotoxins in turkish waterbodies. J. Environ. Prot. Ecol..

[209] Instituto Uruguayo de Normas Técnicas (2008). UNIT 833: Agua Potable—Requisitos.

[210] (2022). Drinking Water Quality.

[211] Ministerio del Ambiente (2017). Decreto Supremo N° 004-2017-MINAM.

[212] Ministerio de Salud (2017). Factores ambientales y antropogénicos que afectan la formación de floraciones de cianobacterias y cianotoxinas. Cianobacterias como determinantes ambientales de la salud. Temas Salud Ambient..

[213] Cantoral E., Asencio A., Aboal M. (2017). Cianotoxinas: Efectos ambientales y sanitarios. Medidas de prevención. Hidrobiológica.

[214] Avendaño A., Arguedas C. (2006). Microcystin in plants that treat water for human consumption in a tropical environment: The Metropolitan Area of Costa Rica. Rev. Biol. Trop..

[215] Secretaría del Ambiente (2002). Resolución Nº 222/02.

[216] Vicepresidencia de Administración del Recurso Hídrico (2022). Informe de Calidad de agua de la Cuenca del Canal 2021.

[217] Flores N.M., Miller T.R., Stockwell J.D. (2018). A global analysis of the relationship between concentrations of microcystins in water and fish. Front. Mar. Sci..

[218] Poste A.E., Hecky R.E., Guildford S.J. (2011). Evaluating microcystin exposure risk through fish consumption. Environ. Sci. Technol..

[219] Shahmohamadloo R.S., Bhavsar S.P., Almirall X.O., Marklevitz S.A., Rudman S.M., Sibley P.K. (2023). Lake Erie fish safe to eat yet afflicted by algal hepatotoxins. Sci. Total Environ..

[220] Weirich C.A., Miller T.R. (2014). Freshwater harmful algal blooms: Toxins and children’s health. Curr. Probl. Pediatr. Adolesc. Health Care.

[221] Lürling M., van der Grinten E. (2003). Life-history characteristics of Daphnia exposed to dissolved microcystin-LR and to the cyanobacterium *Microcystis aeruginosa* with and without microcystins. Environ. Toxicol. Chem. Int. J..

[222] Dao T.S., Cronberg G., Nimptsch J., Do-Hong L.C., Wiegand C. (2010). Toxic cyanobacteria from Tri An Reservoir, Vietnam. Nova Hedwig..

[223] Cerbin S., Kraak M.H.S., De Voogt P., Visser P.M., Van Donk E. (2010). Combined and single effects of pesticide carbaryl and toxic *Microcystis aeruginosa* on the life history of *Daphnia pulicaria*. Hydrobiologia.

[224] Miles C.O., Strand D.A., Rusch J.C., Ballot A., Haande S., Løvberg K.L.E., Vrålstad T., Samdal I.A. (2024). Microcystin profiles in European noble crayfish *Astacus astacus* and water in Lake Steinsfjorden, Norway. Environ. Res..

[225] Berry J.P., Gibbs P.D., Schmale M.C., Saker M.L. (2009). Toxicity of cylindrospermopsin, and other apparent metabolites from *Cylindrospermopsis raciborskii* and *Aphanizomenon ovalisporum*, to the zebrafish (*Danio rerio*) embryo. Toxicon.

[226] Silva C., Anselmo A., Mac’ario I.P.E., de Figueiredo D., Gonçalves F.J.M., Pereira J.L. (2020). The bad against the villain: Suitability of *Corbicula fluminea* as a bioremediation agent towards cyanobacterial blooms. Ecol. Eng..

[227] e Silva F.A., Giani A. (2018). Population dynamic of bloom-forming *Microcystis aeruginosa* in the presence of the invasive bivalve *Limnoperna fortunei*. Harmful Algae.

[228] MacKeigan P.W., Taranu Z.E., Pick F.R., Beisner B.E., Gregory-Eaves I. (2023). Both biotic and abiotic predictors explain significant variation in cyanobacteria biomass across lakes from temperate to subarctic zones. Limnol. Oceanogr..

[229] Anderson L., Fabbro L.D., Cowden K. (2003). Assessment of Blue-Green Algal Toxins in Barramundi, Red Clay and Mussels from Awoonga Dam.

[230] Saker M.L., Metcalf J.S., Codd G.A., Vasconcelos V.M. (2004). Accumulation and depuration of the cyanobacterial toxin cylindrospermopsin in the freshwater mussel Anodonta cygnea. Toxicon.

[231] Jeon B.S., Han J., Kim S.K., Ahn J.H., Oh H.C., Park H.D. (2015). An overview of problems cyanotoxins produced by cyanobacteria and the solutions thereby. J. Korean Soc. Environ. Eng..

[232] Chen J., Xie P., Li L., Xu J. (2009). First Identification of the Hepatotoxic Microcystins in the Serum of a Chronically Exposed Human Population Together with Indication of Hepatocellular Damage. Toxicol. Sci..

[233] Sasner J.J., Ikawa M., Foxall T.L. (1984). Studies on aphanizomenon and microcystis toxins. Seaf. Toxins.

[234] Negri A.P., Jones G.J. (1995). Bioaccumulation of paralytic shellfish poisoning (PSP) toxins from the cyanobacterium *Anabaena circinalis* by the freshwater mussels *Alathrya condola*. Toxicon.

[235] Karlson A.M.L., Mozūraitis R. (2011). Deposit-feeders accumulate the cyanobacterial toxin nodularin. Harmful Algae.

[236] Strogyloudi E., Giannakourou A., Legrand C., Ruehl A., Granéli E. (2006). Estimating the accumulation and transfer of *Nodularia spumigena* toxins by the blue mussel *Mytilus edulis*: An appraisal from culture and mesocosm experiments. Toxicon.

[237] España Amórtegui J.C., Pekar H., Chico Retrato M.D., Persson M., Karlson B., Bergquist J., Zuberovic-Muratovic A. (2023). LC-MS/MS Analysis of Cyanotoxins in Bivalve Mollusks-Method Development, Validation and First Evidence of Occurrence of Nodularin in Mussels (*Mytilus edulis*) and Oysters (*Magallana gigas*) from the West Coast of Sweden. Toxins.

[238] Sipiä V., Kankaanpää H.T., Flinkman J., Lahti K., Meriluoto J.A.O. (2001). Time-dependent accumulation of cyanobacterial hepatotoxins in flounders (*Platichthys flesus*) and Mussels (*Mytilus edulis*) from the Northern Baltic Sea. Environ. Toxicol..

[239] Kankaanpää H.T., Leiniö S., Olin M., Sjövall O., Meriluoto J., Lehtonen K.K. (2007). Accumulation and depuration of cyanobacterial toxin nodularin and biomarker responses in the mussel *Mytilus edulis*. Chemosphere.

[240] White S.H., Duivenvoorden L.J., Fabbro L.D., Eaglesham G.K. (2006). Influence of intracellular toxin concentrations on cylindrospermopsin bioaccumulation in a freshwater gastropod (*Melanoidestuberculata*). Toxicon.

[241] Vareli K., Zarali E., Zacharioudakis G.S., Vagenas G., Varelis V., Pilidis G., Briasoulis E., Sainis I. (2012). Microcystin producing cyanobacterial communities in Amvrakikos Gulf (Mediterranean Sea, NW Greece) and toxin accumulation in mussels (*Mytilus galloprovincialis*). Harmful Algae.

[242] Preece E.P., Moore B.C., Joan Hardy F. (2015). Transfer of microcystin from freshwater lakes to Puget Sound, WA and toxin accumulation in marine mussels (*Mytilus trossulus*). Ecotoxicol. Environ. Saf..

[243] Wood S., Phillips N.R., Winton M.D., Gibbs M.M. (2012). Consumption of benthic cyanobacterial mats and nodularin-R accumulation in freshwater crayfish (*Paranephrops planifrons*) in Lake Tikitapu (Rotorua, New Zealand). Harmful Algae.

[244] Saker M.L., Eaglesham G.K. (1999). The accumulation of cylindrospermopsin from the cyanobacterium *Cylindrospermopsis raciborskii* in tissues of the Redclaw crayfish *Cherax quadricarinatus*. Toxicon.

[245] Zhang D., Xie P., Chen J., Dai M., Qiu T., Liu Y., Liang G. (2009). Determination of microcystin-LR and its metabolites in snail (*Bellamya aeruginosa*), shrimp (*Macrobrachium nipponensis*) and silver carp (*Hypophthalmichthys molitrix*) from Lake Taihu, China. Chemosphere.

[246] Chen J., Xie P., Guo L., Zheng L., Ni L. (2005). Tissue distributions and seasonal dynamics of the hepatotoxic microcystins-LR and -RR in a freshwater snail (*Bellamya aeruginosa*) from a large shallow, eutrophic lake of the subtropical China. Environ. Pollut..

[247] Gérard C., Poullain V., Lance E., Acou A., Brient L., Carpentier A. (2009). Influence of toxic cyanobacteria on community structure and microcystin accumulation of freshwater molluscs. Environ. Pollut..

[248] Lance E., Neffling M.R., Gérard C., Meriluoto J., Bormans M. (2010). Accumulation of free and covalently bound microcystins in tissues of *Lymnaea stagnalis* (Gastropoda) following toxic cyanobacteria or dissolved microcystin-LR exposure. Environ. Pollut..

[249] Zhang J., Wang Z., Song Z., Xie Z., Li L., Song L. (2012). Bioaccumulation of microcystins in two freshwater gastropods from a cyanobacteria-bloom plateau lake, Lake Dianchi. Environ. Pollut..

[250] Gérard C., Lance E. (2019). Decline of freshwater gastropods exposed to recurrent interacting stressors implying cyanobacterial proliferations and droughts. Aquat. Ecol..

[251] Berry J.P., Lind O. (2010). First evidence of “paralytic shellfish toxins” and cylindrospermopsin in a Mexican freshwater system, Lago Catemaco, and apparent bioaccumulation of the toxins in “tegogolo” snails (*Pomacea patula catemacensis*). Toxicon.

[252] Xie L., Yokoyama A., Nakamura K., Park H. (2007). Accumulation of microcystins in various organs of the freshwater snail *Sinotaia histrica* and three fishes in a temperate lake, the eutrophic Lake Suwa, Japan. Toxicon.

[253] Engström-öst J., Lehtinienmi M., Green S., Kozlowsky-Suzuki B., Viitassalo M. (2002). Does cyanobacterial toxin accumulate in mysid shrimps and fish via copepods?. J. Exp. Mar. Biol. Ecol..

[254] Garcia A.C., Bargu S., Dash P., Rabalais N.N., Sutor M.M., Morrison W., Walker N.D. (2010). Evaluating the potential risk of microcystins to blue crab (*Callinectes sapidus*) fisheries and human health in a eutrophic estuary. Harmful Algae.

[255] Berry J.P., Jaja-Chimedza A., Dávalos-Lind L., Lind O. (2011). Apparent bioaccumulation of cylindrospermopsin and paralytic shellfish toxins by finfish in Lake Catemaco (Veracruz, Mexico). Food Addit. Contam..

[256] Kankaanpää H.T., Holliday J., Schröder H., Goddard T.J., Von Fister R., Carmichael W.W. (2005). Cyanobacteria and prawn farming in northern New South Wales, Australia-a case study on cyanobacteria diversity and hepatotoxin bioaccumulation. Toxicol. Appl. Pharmacol..

[257] Chen J., Zhang D., Xie P., Wang Q., Ma Z. (2009). Simultaneous determination of microcystin contaminations in various vertebrates (fish, turtle, duck and water bird) from a large eutrophic Chinese lake, Lake Taihu, with toxic Microcystis blooms. Sci. Total Environ..

[258] Pawlik-Skowrońska B., Toporowska M., Rechulicz J. (2012). Simultaneous accumulation of anatoxin-a and microcystins in three fish species indigenous to lakes affected by cyanobacterial blooms. Oceanol. Hydrobiol. Stud..

[259] Sipiä V., Kankaanpää H., Peltonen H., Vinni M., Meriluoto J. (2007). Transfer of nodularin to three-spined stickleback (*Gasterosteus aculeatus* L.), herring (*Clupea harengus* L.), and salmon (*Salmo salar* L.) in the northern Baltic Sea. Ecotoxicol. Environ. Saf..

[260] Clemente Z., Busato R.H., Oliveira Ribeiro C.A., Cestari M.M., Ramsdorf W.A., Magalhães V.F., Wosiack A.C., Silva de Assis H.C. (2010). Analyses of paralytic shellfish toxins and biomarkers in a southern Brazilian reservoir. Toxicon.

[261] Roegner A.F., Corman J.R., Sitoki L.M., Kwena Z.A., Ogari Z. (2023). Impacts of algal blooms and microcystins in fish on small-scalefishers in Winam Gulf, Lake Victoria: Implications for health and livelihood. Ecol. Soc..

[262] Scarlett K.R., Kim S., Lovin L.M., Chatterjee S., Scott J.T., Brooks B.W. (2020). Global scanning of cylindrospermopsin: Critical review and analysis of aquatic occurrence, bioaccumulation, toxicity and health hazards. Sci. Total Environ..

[263] Cadel-Six S., Moyenga D., Magny S., Trotereau S., Edery M., Krys S. (2014). Detection of free and covalently bound microcystins in different tissues (liver, intestines, gills, and muscles) of rainbow trout (*Oncorhynchus mykiss*) by liquid chromatography-tandem mass spectrometry: Method characterization. Environ. Pollut..

[264] Mohamed Z.A., Bakr A. (2018). Concentrations of cylindrospermopsin toxin in water and tilapia fish of tropical fishponds in Egypt, and assessing their potential risk to human health. Environ. Sci. Pollut. Res..

[265] Wilson A.E., Gossiaux D.C., Höök T.O., Berry J.P., Landrum P.F., Dyble J., Guildford S.J. (2008). Evaluation of the human health threat associated with the hepatotoxin microcystin in the muscle and liver tissues of yellow perch (*Perca flavescens*). Can. J. Fish. Aquat. Sci..

[266] Ibelings B.W., Bruning K., de Jonge J., Wolfstein K., Dionisio Pires L.M., Postma J., Burger T. (2005). Distribution of Microcystins in a Lake Foodweb: No Evidence for Biomagnification. Microb. Ecol..

[267] Sipiä V., Sjövall O., Valtonen T., Barnaby D.L., Codd G.A., Metcalf J.S., Kilpi M., Mustonen O., Meriluoto J.A.O. (2006). Analysis of nodularin-R in eider (*Somateria mollissima*), roach (*Rutilus rutilus* L.), and flounder (*Platichthys flesus* L.) liver and muscle samples from the western Gulf of Finland, northern Baltic Sea. Environ. Toxicol. Chem..

[268] Kankaanpää H.T., Vuorinen P.J., Sipiä V., Keinänen M. (2002). Acute effects and bioaccumulation of nodularin in sea trout (*Salmo trutta* m. *trutta* L.) exposed orally to *Nodularia spumigena* under laboratory conditions. Aquat. Toxicol..

[269] Soares R.M., Magalhães V.F., Azevedo S. (2004). Accumulation and depuration of microcystins (cyanobacteria hepatotoxins) in *Tilapia rendalli* (Cichlidae) under laboratory conditions. Aquat. Toxicol..

[270] Acou A., Robinet T., Lance E., Gérard C., Mounaix B., Brient L., Le Rouzic B., Feunteun E. (2008). Evidence of silver eels contamination by microcystin-LR at the onset of their seaward migration: What consequences for breeding potential?. J. Fish. Biol..

[271] Stewart I., Eaglesham J.K., McGregor G.B., Chong R., Seawright A.A., Wickramasinghe W.A., Sadler R., Hunt L., Graham G. (2012). First Report of a Toxic *Nodularia spumigena* (Nostocales/Cyanobacteria) Bloom in Sub-Tropical Australia. II. Bioaccumulation of Nodularin in Isolated Populations of Mullet (Mugilidae). Int. J. Environ. Res. Public Health.

[272] Mazur-Marzec H., Tymińska A., Szafranek J., Pliński M. (2007). Accumulation of nodularin in sediments, mussels, and fish from the Gulf of Gdańsk, southern Baltic Sea. Environ. Toxicol..

[273] Sipiä V., Kankaanpää H.T., Pflugmacher S., Flinkman J., Furey A., James K.J. (2002). Bioaccumulation and Detoxication of Nodularin in Tissues of Flounder (*Platichthys flesus*), Mussels (*Mytilus edulis*, *Dreissena polymorpha*), and Clams (*Macoma balthica*) from the Northern Baltic Sea. Ecotoxicol. Environ. Saf..

[274] Persson K.J., Legrand C., Olsson T. (2009). Detection of nodularin in European flounder (*Platichthys flesus*) in the west coast of Sweden: Evidence of nodularin mediated oxidative stress. Harmful Algae.

[275] Li Y., Chen J.A., Zhao Q., Pu C., Qiu Z., Zhang R., Shu W. (2011). A cross-sectional investigation of chronic exposure to microcystin in relationship to childhood liver damage in the Three Gorges Reservoir Region, China. Environ. Health Perspect..

[276] Azevedo S., Carmichael W.W., Jochimsen E.M., Rinehart K.L., Lau S., Shaw G.R., Eaglesham G.K. (2002). Human intoxication by microcystins during renal dialysis treatment in Caruaru-Brazil. Toxicology.

[277] Hilborn E.D., Carmichael W.W., Yuan M., Azevedo S.M.F.D.O.E. (2005). A simple colorimetric method to detect biological evidence of human exposure to microcystins. Toxicon.

[278] Soares R.M., Yuan M., Servaites J.C., Delgado A., Magalhães V.F., Hilborn E.D., Carmichael W.W., Azevedo S. (2006). Sublethal exposure from microcystins to renal insufficiency patients in Rio de Janeiro, Brazil. Environ. Toxicol..

[279] Dreher T.W., Collart L.P., Mueller R.S., Halsey K.H., Bildfell R.J., Schreder P., Sobhakumari A., Ferry R. (2019). *Anabaena*/*Dolichospermum* as the source of lethal microcystin levels responsible for a large cattle toxicosis event. Toxicon X.

[280] Badar M., Batool F., Shah Khan S., Khokhar I., Qamar M.K., Yasir C. (2017). Effects of microcystins toxins contaminated drinking water on hepatic problems in animals (cows and buffalos) and toxins removal chemical method. Buffalo Bull..

[281] Handeland K., Østensvik Ø. (2010). Microcystin poisoning in roe deer (*Capreolus capreolus*). Toxicon.

[282] Foss A.J., Aubel M.T., Gallagher B., Mettee N., Miller A., Fogelson S.B. (2019). Diagnosing Microcystin Intoxication of Canines: Clinicopathological Indications, Pathological Characteristics, and Analytical Detection in Postmortem and Antemortem Samples. Toxins.

[283] Turner A.D., Turner F.R.I., White M., Hartnell D., Crompton C.G., Bates N., Egginton J., Branscombe L., Lewis A.M., Maskrey B.H. (2022). Confirmation Using Triple Quadrupole and High-Resolution Mass Spectrometry of a Fatal Canine Neurotoxicosis following Exposure to Anatoxins at an Inland Reservoir. Toxins.

[284] Fredrickson A., Richter A., Perri K.A., Manning S.R. (2023). First Confirmed Case of Canine Mortality Due to Dihydroanatoxin-a in Central Texas, USA. Toxins.

[285] Faassen E.J., Harkema L., Begeman L., Lurning M. (2012). First report of (homo)anatoxin-a and dog neurotoxicosis after ingestion of benthic cyanobacteria in The Netherlands. Toxicon.

[286] Gugger M., Lenoir S., Berger C., Ledreux A., Druart J.C., Humbert J.F., Guette C., Bernard C. (2005). First report in a river in France of the benthic cyanobacterium *Phormidium favosum* producing anatoxin-a associated with dog neurotoxicosis. Toxicon.

[287] McCarron P., Rafuse C., Scott S., Lawrence J.E., Bruce M.R., Douthwright E., Murphy C., Reith M., Beach D.G. (2023). Anatoxins from benthic cyanobacteria responsible for dog mortalities in New Brunswick, Canada. Toxicon.

[288] Brown A.O., Foss A., Miller M.A., Gibson Q.A. (2018). Detection of cyanotoxins (microcystins/nodularins) in livers from estuarine and coastal bottlenose dolphins (*Tursiops truncatus*) from Northeast Florida. Harmful Algae.

[289] Greer B., Meneely J.P., Elliott C.T. (2018). Uptake and accumulation of Microcystin-LR based on exposure through drinking water: An animal model assessing the human health risk. Sci. Rep..

[290] White S.H., Duivenvoorden L.J., Fabbro L.D., Eaglesham G.K. (2007). Mortality and toxin bioaccumulation in *Bufo marinus* following exposure to *Cylindrospermopsis raciborskii* cell extracts and live cultures. Environ. Pollut..

[291] Su R.C., Meyers C.M., Warner E.A., Garcia J.A., Refsnider J.M., Lad A., Breidenbach J.D., Modyanov N., Malhotra D., Haller S.T. (2020). Harmful algal bloom toxicity in *Lithobates catesbeiana* tadpoles. Toxins.

[292] Nasri H., El Herry S., Bouaïcha N. (2008). First reported case of turtle deaths during a toxic *Microcystis* spp. bloom in Lake Oubeira, Algeria. Ecotoxicol. Environ. Saf..

[293] Zhang D., Yuan L., Zhang L., Qiu T., Liao Q., Xiang J., Luo L., Xiong X. (2022). Pathological and biochemical characterizations of microcystin-LR-induced liver and kidney damage in chickens after acute exposure. Toxicon.

[294] Sipiä V., Karlsson K.M., Meriluoto J.A.O., Kankaanpää H.T. (2009). Eiders (*Somateria mollissima*) obtain nodularin, a cyanobacterial hepatotoxin, in baltic sea food web. Environ. Toxicol. Chem..

[295] Krienitz L., Ballot A., Kotut K., Wiegand C., Pütz S., Metcalf J.S., Codd G.A., Pflugmacher S. (2003). Contribution of hot spring cyanobacteria to the mysterious deaths of Lesser Flamingos at Lake Bogoria, Kenya. FEMS Microbiol. Ecol..

[296] Alonso-Andicoberry C., García-Villada L., López-Rodas V., Costas E. (2002). Catastrophic mortality of flamingos in a Spanish national park caused by cyanobacteria. Vet. Rec..

[297] Pflugmacher S. (2002). Possible allelopathic effects of cyanotoxins, with reference to microcystin-LR, in aquatic ecosystems. Environ. Toxicol..

[298] Biré R., Bertin T., Dom I., Hort V., Schmitt C., Diogène J., Lemée R., De Haro L., Nicolas M. (2020). First Evidence of the Presence of Anatoxin-a in Sea Figs Associated with Human Food Poisonings in France. Mar. Drugs.

[299] Sabatini S.E., Brena B.M., Luquet C.M., San Julian M., Pirez M., de Molina M. (2011). Microcystin accumulation and antioxidant responses in the freshwater clam *Diplodon chilensis* patagonicus upon subchronic exposure to toxic *Microcystis aeruginosa*. Ecotoxicol. Environ. Saf..

[300] Pflugmacher S., Wiegand C., Oberemm A., Beattie K.A., Krause E., Codd G.A., Steinberg C.E. (1998). Identification of an enzymatically formed glutathione conjugate of the cyanobacterial hepatotoxin microcystin-LR: The first step of detoxication. Biochim. Biophys. Acta BBA-Gen. Subj..

[301] Peuthert A., Wiegand C. (2024). Glutathione involvement in the biotransformation of microcystins in *Dreissena polymorpha*. Poster Abstract, Proceedings of the 6th International Conference on Toxic Cyanobacteria (ICTC), Bergen, Norway, 21–27 June 2004.

[302] Grosse Y., Baan R., Straif K., Secretan B., El Ghissassi F., Cogliano V. (2006). Carcinogenicity of nitrate, nitrite, and cyanobacterial peptide toxins. Lancet Oncol..

[303] Ibelings B.W., Chorus I. (2007). Accumulation of cyanobacterial toxins in freshwater “seafood” and its consequences for public health: A review. Environ. Pollut..

[304] Butler N., Carlisle J., Linville R. (2012). Toxicological Summary and Suggested Action Levels to Reduce Potential Adverse Health Effects of Six Cyanotoxins.

[305] Williams D.E., Dawe S.C., Kent M.L., Andersen R.J., Craig M., Holmes C.F.B. (1997). Bioaccumulation and clearance of microcystins from salt water mussels, *Mytilus edulis*, and in vivo evidence for covalently bound microcystins in mussel tissues. Toxicon.

[306] Miles C.O., Sandvik M., Nonga H.E., Ballot A., Wilkins A.L., Rise F., Jaabaek A.H., Loader J.I. (2016). Conjugation of microcystins with thiols is reversible: Base-catalyzed deconjugation for chemical analysis. Chem. Res. Toxicol..

[307] Shahmohamadloo R.S., Frenken T., Rudman S.M., Ibelings B.W., Trainer V.L. (2023). Diseases and disorders in fish due to harmful algal blooms. Climate Change on Diseases and Disorders of Finfish in Cage Culture.

[308] Ernst B., Hoeger S.J., O’Brien E., Dietrich D.R. (2006). Oral toxicity of the microcystin-containing cyanobacterium *Planktothrix rubescens* in European whitefish (*Coregonus lavaretus*). Aquat Toxicol..

[309] de Lima Pinheiro M.M., Santos B.L.T., Dantas Filho J.V., Pedroti V.P., Cavali J., Dos Santos R.B., Oliveira Carreira Nishiyama A.C., Guedes E.A.C., de Vargas Schons S. (2023). First monitoring of cyanobacteria and cyanotoxins in freshwater from fish farms in Rondônia state, Brazil. Heliyon.

[310] Zamora-Barrios C.A., Nandini S., Sarma S. (2019). Bioaccumulation of microcystins in seston, zooplankton and fish: A case study in Lake Zumpango, Mexico. Environ. Pollut..

[311] Berry J.P., Lee E., Walton K., Wilson A.E., Bernal-Brooks F. (2011). Bioaccumulation of microcystins by fish associated with a persistent cyanobacterial bloom in Lago de Patzcuaro (Michoacan, Mexico). Environ. Toxicol. Chem..

[312] Dolamore B., Puddick J., Wood S. (2016). Accumulation of nodularin in New Zealand shortfin eel (*Anguilla australis*): Potential consequences for human consumption. N. Z. J. Mar. Freshw. Res..

[313] Bukaveckas P.A., Lesutienė J., Gasiūnaitė Z.R., Ložys L., Olenina I., Pilkaitytė R., Pūtys Ž., Tassone S., Wood J. (2017). Microcystin in aquatic food webs of the Baltic and Chesapeake Bay regions. Estuar. Coast. Shelf Sci..

[314] Díez-Quijada L., Medrano-Padial C., Llana-Ruiz-Cabello M., Cătunescu G.M., Moyano R., Risalde M.A., Cameán A.M., Jos A. (2020). Cylindrospermopsin-microcystin-LR combinations may induce genotoxic and histopathological damage in rats. Toxins.

[315] Kruk C., Martínez A., de la Escalera G.M., Trinchin R., Manta G., Segura A.M., Piccini C., Brena B., Yannicelli B., Fabiano G. (2021). Rapid freshwater discharge on the coastal ocean as a mean of long distance spreading of an unprecedented toxic cyanobacteria bloom. Sci. Total Environ..

[316] Preece E.P., Hardy F.J., Moore B.C., Bryan M. (2017). A review of microcystin detections in Estuarine and Marine waters: Environmental implications and human health risk. Harmful Algae.

[317] Mulvenna V., Dale K., Priestly B., Mueller U., Humpage A., Shaw G., Allinson G., Falconer I. (2012). Health risk assessment for cyanobacterial toxins in seafood. Int. J. Environ. Res. Public Health.

[318] Gibble C.M., Peacock M.B., Kudela R.M. (2016). Evidence of freshwater algal toxins in marine shellfish: Implications for human and aquatic health. Harmful Algae.

[319] Fristachi A., Sinclair J.L., Hall S., Berkman J.A.H., Boyer G., Burkholder J., Burns J., Carmichael W., DuFour A., Frazier W. (2008). Occurrence of cyanobacterial harmful algal blooms workgroup report. Cyanobacterial Harmful Algal Blooms: State of the Science and Research Needs.

[320] Vuorinen P.J., Sipiä V.O., Karlsson K., Keinänen M., Furey A., Allis O., James K., Perttilä U., Rimaila-Pärnänen E., Meriluoto J.A.O. (2009). Accumulation and effects of nodularin from a single and repeated oral doses of cyanobacterium *Nodularia spumigena* on flounder (*Platichthys flesus* L.). Arch. Environ. Contam. Toxicol..

[321] Al-Tebrineh J., Mihali T.K., Pomati F., Neilan B.A. (2010). Detection of saxitoxin-producing cyanobacteria and *Anabaena circinalis* in environmental water blooms by quantitative PCR. Appl. Environ. Microbiol..

[322] Nauman C., Stanislawczyk K., Reitz L.A., Chaffin J.D. (2024). The spatiotemporal distribution of potential saxitoxin-producing cyanobacteria in western Lake Erie. J. Great Lakes Res..

[323] Jeon Y., Li L., Bhatia M., Ryu H., Santo Domingo J.W., Brown J., Goetz J., Seo Y. (2024). Impact of harmful algal bloom severity on bacterial communities in a full-scale biological filtration system for drinking water treatment. Sci. Total Environ..

[324] Kim J., Lee G., Han S., Kim M.J., Shin J.H., Lee S. (2023). Microbial communities in aerosol generated from cyanobacterial bloom-affected freshwater bodies: An exploratory study in Nakdong River, South Korea. Front. Microbiol..

[325] Montuori E., De Luca D., Penna A., Stalberga D., Lauritano C. (2023). *Alexandrium* spp.: From Toxicity to Potential Biotechnological Benefits. Mar. Drugs.

[326] García C., Pérez F., Contreras C., Figueroa D., Barriga A., López-Rivera A., Araneda O.F., Contreras H.R. (2015). Saxitoxins and okadaic acid group: Accumulation and distribution in invertebrate marine vectors from Southern Chile. Food Addit. Contam. Part A Chem. Anal. Control Expo. Risk Assess..

[327] Seguel M., Molinet C., Díaz M., Álvarez G., García C., Marín A., Millanao M.O., Díaz P.A. (2023). Paralytic shellfish toxins in the gastropod *Concholepas concholepas*: Variability, toxin profiles and mechanisms for toxicity reduction. Mar. Drugs.

[328] Zamorano R., Marín M., Cabrera F., Figueroa D., Contreras C., Barriga A., Lagos N., García C. (2013). Determination of the Variability of both Hydrophilic and Lipophilic Toxins in Endemic Bivalves and Carnivores from Austral Pacific’s Fjords. Food Addit. Contam. Part A.

[329] Hong Z., Chen X., Hu J., Chang X., Qian Y. (2024). Adverse effects of *Microcystis aeruginosa* exudates on the filtration, digestion, and reproduction organs of benthic bivalve *Corbicula fluminea*. Sci. Rep..

[330] Álvarez G., Díaz P.A., Godoy M., Araya M., Ganuza I., Pino R., Álvarez F., Rengel J., Hernández C., Uribe E. (2019). Paralytic Shellfish Toxins in Surf Clams *Mesodesma donacium* during a Large Bloom of *Alexandrium catenella* Dinoflagellates Associated to an Intense Shellfish Mass Mortality. Toxins.

[331] Cao A., Vilariño N., Botana L.M. (2024). The problem of cyanotoxins in freshwaters: From climate change to detoxification. Freshwater Researches: Fundamentals, Trends and Perspectives.

[332] Anderson M., Valera M., Schnetzer A. (2023). Co-occurrence of freshwater and marine phycotoxins: A record of microcystins and domoic acid in Bogue Sound, North Carolina (2015 to 2020). Harmful Algae.

[333] Bormans M., Amzil Z., Mineaud E., Brient L., Savar V., Robert E., Lance E. (2019). Demonstrated transfer of cyanobacteria and cyanotoxins along a freshwater-marine continuum in France. Harmful Algae.

[334] Overlingė D., Kataržytė M., Vaičiūtė D., Gyraite G., Gečaitė I., Jonikaitė E., Mazur-Marzec H. (2020). Are there concerns regarding cHAB in coastal bathing waters affected by freshwater-brackish continuum?. Mar. Pollut. Bull..

[335] Ibelings B.W., Havens K.E. (2008). Cyanobacterial toxins: A qualitative meta-analysis of concentrations, dosage and effects in freshwater, estuarine and marine biota. Cyanobacterial Harmful Algal Blooms: State of the Science and Research Needs.

[336] Jia J., Luo W., Lu Y., Giesy J.P. (2014). Bioaccumulation of microcystins (MCs) in four fish species from Lake Taihu, China: Assessment of risks to humans. Sci. Total Environ..

[337] Carmichael W.W. (1997). The cyanotoxins. Adv. Bot. Res..

[338] Griffiths D.J., Saker M.L. (2003). The Palm Island mystery disease 20 years on: A review of research on the cyanotoxin cylindrospermopsin. Environ. Toxicol. Int. J..

[339] Foss A.J., Butt J., Aubel M.T. (2008). Benthic periphyton from Pennsylvania, USA is a source for both hepatotoxins (microcystins/nodularin) and neurotoxins (anatoxin-a/homoanatoxin-a). Toxicon.

[340] Cordeiro-Araújo M.K., Chia M.A., Bittencourt-Oliveira M.d.C. (2017). Potential human health risk assessment of cylindrospermopsin accumulation and depuration in lettuce and arugula. Harmful Algae.

[341] Haida M., El Khalloufi F., Mugani R., Essadki Y., Campos A., Vasconcelos V., Oudra B. (2024). Microcystin Contamination in Irrigation Water and Health Risk. Toxins.

[342] Metcalf J.S., Banack S.A., Lindsay J., Morrison L.F., Cox P.A., Codd G.A. (2008). Co-occurrence of beta-N-methylamino-L-alanine, a neurotoxic amino acid with other cyanobacterial toxins in British waterbodies, 1990–2004. Environ. Microbiol..

[343] Codd G., Bell S., Kaya K., Ward C., Beattie K., Metcalf J. (1999). Cyanobacterial toxins, exposure routes and human health. Eur. J. Phycol..

[344] Redouane E.M., Tazart Z., Lahrouni M., Mugani R., Elgadi S., Zine H., El Amrani Zerrifi S., Haida M., Martins J.C., Campos A. (2023). Health risk assessment of lake water contaminated with microcystins for fruit crop irrigation and farm animal drinking. Environ. Sci. Pollut. Res..

[345] Hereman T.C., Bittencourt-Oliveira M. (2012). Bioaccumulation of Microcystins in Lettuce. J. Phycol..

[346] Bullerjahn G.S., McKay R.M., Davis T.W., Baker D.B., Boyer G.L., D’Anglada L.V., Doucette G.J., Ho J.C., Irwin E.G., Kling C.L. (2016). Global solutions to regional problems: Collecting global expertise to address the problem of harmful cyanobacterial blooms. A Lake Erie case study. Harmful Algae.

[347] Svirčev Z., Drobac D., Tokodi N., Mijović B., Codd G.A., Meriluoto J. (2017). Toxicology of microcystins with reference to cases of human intoxications and epidemiological investigations of exposures to cyanobacteria and cyanotoxins. Arch. Toxicol..

[348] Manganelli M., Testai E., Tazart Z., Scardala S., Codd G.A. (2023). Co-occurrence of taste and odor compounds and cyanotoxins in cyanobacterial blooms: Emerging risks to human health?. Microorganisms.

[349] Plaas H.E., Paerl H.W. (2020). Toxic cyanobacteria: A growing threat to water and air quality. Environ. Sci. Technol..

[350] Ressom R. (1994). Health Effects of Toxic Cyanobacteria (Blue-Green Algae).

[351] Kondo F., Matsumoto H., Yamada S., Ishikawa N., Ito E., Nagata S., Ueno Y., Suzuki M., Harada K. (1996). Detection and identification of metabolites of microcystins formed in vivo in mouse and rat livers. Chem. Res. Toxicol..

[352] Hilborn E.D., Carmichael W.W., Soares R.M., Yuan M., Servaites J.C., Barton H.A., Azevedo S.M.F.O. (2007). Serologic evaluation of human microcystin exposure. Environ. Toxicol..

[353] Blyth S. (1980). Palm Island mystery disease. Med. J. Aust..

[354] Hawkins P.R., Runnegar M.T., Jackson A.R., Falconer I. (1985). Severe hepatotoxicity caused by the tropical cyanobacterium (blue-green alga) *Cylindrospermopsis raciborskii* (Woloszynska) Seenaya and Subba Raju isolated from a domestic water supply reservoir. Appl. Environ. Microbiol..

[355] Mchau G., Machunda R., Kimanya M., Makule E., Gong Y., Mpolya E., Meneely J., Elliott C., Greer B. (2021). First Report of the Co-occurrence of Cylindrospermopsin, Nodularin and Microcystins in the Freshwaters of Lake Victoria, Tanzania. Expo Health.

[356] Behm D. (2003). Coroner cites algae in teen’s death. Milwaukee J. Sentin..

[357] Giannuzzi L., Sedan D., Echenique R., Andrinolo D. (2011). An acute case of intoxication with cyanobacteria and cyanotoxins in recreational water in Salto Grande Dam, Argentina. Mar. Drugs.

[358] Vidal F., Sedan D., D’Agostino D., Cavalieri M.L., Mullen E., Parot Varela M.M., Flores C., Caixach J., Andrinolo D. (2017). Recreational exposure during algal bloom in Carrasco Beach, Uruguay: A liver failure case report. Toxins.

[359] Bloch R.A., Beuhler M.C., Hilborn E.D., Faulkner G., Rhea S. (2024). Epidemiologic and clinical features of cyanobacteria harmful algal bloom exposures reported to the National Poison Data System, United States, 2010–2022: A descriptive analysis. Environ. Health.

[360] Kubickova B., Babica P., Hilscherová K., Šindlerová L. (2019). Effects of cyanobacterial toxins on the human gastrointestinal tract and the mucosal innate immune system. Environ. Sci. Eur..

[361] Nielsen M.C., Jiang S.C. (2020). Can cyanotoxins penetrate human skin during water recreation to cause negative health effects?. Harmful Algae.

[362] Kholafazad Kordasht H., Hassanpour S., Baradaran B., Nosrati R., Hashemzaei M., Mokhtarzadeh A., de la Guardia M. (2020). Biosensing of microcystins in water samples; recent advances. Biosens. Bioelectron..

[363] French B.W., Kaul R., George J., Haller S.T., Kennedy D.J., Mukundan D. (2023). A Case Series of Potential Pediatric Cyanotoxin Exposures Associated with Harmful Algal Blooms in Northwest Ohio. Infect. Dis. Rep..

[364] Drobac Backović D., Tokodi N., Marinović Z., Lujić J., Dulić T., Simić S.B., Đorđević N.B., Kitanović N., Šćekić I., Urbányi B. (2021). Cyanobacteria, cyanotoxins, and their histopathological effects on fish tissues in Fehérvárcsurgó reservoir, Hungary. Environ. Monit. Assess..

[365] Gaget V., Humpage A.R., Huang Q., Monis P., Brookes J.D. (2017). Benthic cyanobacteria: A source of cylindrospermopsin and microcystin in Australian drinking water reservoirs. Water Res..

[366] Pham T.L., Shimizu K., Dao T.S., Motoo U. (2017). First report on free and covalently bound microcystins in fish and bivalves from Vietnam: Assessment of risks to humans. Environ. Toxicol. Chem..

[367] Díez-Quijada L., Guzmán-Guillén R., Prieto Ortega A.I., Llana-Ruíz-Cabello M., Campos A., Vasconcelos V., Jos A., Cameán A.M. (2018). New method for simultaneous determination of microcystins and cylindrospermopsin in vegetable matrices by SPE-UPLC-MS/MS. Toxins.

[368] Zhang B., Zhang Y., Downing A., Niu Y. (2011). Distribution and composition of cyanobacteria and microalgae associated with biological soil crusts in the Gurbantunggut Desert, China. Arid Land Res. Manag..

[369] Ibelings B.W., Kurmayer R., Azevedo S.M., Wood S.A., Chorus I., Welker M. (2021). Understanding the occurrence of cyanobacteria and cyanotoxins. Toxic Cyanobacteria in Water.

[370] Plaas H.E., Paerl R.W., Baumann K., Karl C., Popendorf K.J., Barnard M.A., Chang N.Y., Curtis N.P., Huang H., Mathieson O.L. (2022). Harmful cyanobacterial aerosolization dynamics in the airshed of a eutrophic estuary. Sci. Total Environ..

[371] Fleming E.D., Bebout B.M., Castenholz R.W. (2021). Photochemical changes during rehydration of an intertidal cyanobacterial mat exposed to variations in salinity and light intensity. Microbiology.

[372] Reichwaldt E.S., Stone D., Barrington D.J., Sinang S.C., Ghadouani A. (2016). Development of toxicological risk assessment models for acute and chronic exposure to pollutants. Toxins.

[373] Walls J.T., Wyatt K.H., Doll J.C., Rubenstein E.M., Rober A.R. (2018). Hot and toxic: Temperature regulates microcystin release from cyanobacteria. Sci. Total Environ..

[374] Corbel S., Mougin C., Bouaïcha N. (2014). Cyanobacterial toxins: Modes of actions, fate in aquatic and soil ecosystems, phytotoxicity and bioaccumulation in agricultural crops. Chemosphere.

[375] Glibert P.M., Maranger R., Sobota D.J., Bouwman L. (2014). The Haber Bosch-harmful algal bloom (HB-HAB) link. Environ. Res. Lett..

[376] Lyu K., Gu L., Li B., Lu Y., Wu C., Guan H., Yang Z. (2016). Stress-responsive expression of a glutathione S-transferase(delta) gene in waterflea *Daphnia magna* challenged by microcystin-producing and microcystin-free *Microcystis aeruginosa*. Harmful Algae.

[377] Olano H., Martigani F., Somma A., Aubriot L. (2019). Wastewater discharge with phytoplankton may favor cyanobacterial development in the main drinking water supply river in Uruguay. Environ. Monit. Assess..

[378] Portner H.O., Masson-Delmotte V., Roberts D., Zhai P. (2019). IPCC Special Report on the Ocean and Crysphere in a Changing Climate.

[379] Chen N., Liu L., Chen M.S., Li Y.F., Xing X.G., Lv Y.Y. (2016). Effects of benthic bioturbation on phytoplankton in eutrophic water: A laboratory experiment. Fundam. Appl. Limnol..

[380] Guo Y., Meng H., Zhao S., Wang Z., Zhu L., Deng D., Liu J., He H., Xie W., Wang G. (2023). How does *Microcystis aeruginosa* respond to elevated temperature?. Sci. Total Environ..

[381] Jiang M., Zheng Z. (2018). Effects of multiple environmental factors on the growth and extracellular organic matter production of *Microcystis aeruginosa*: A central composite design response surface model. Environ. Sci. Pollut. Res..

[382] Martin R.M., Moniruzzaman M., Stark G.F., Gann E.R., Derminio D.S., Wei B., Hellweger F.L., Pinto A., Boyer G.L., Wilhelm S.W. (2020). Episodic decrease in temperature increases mcy gene transcription and cellular microcystin in continuous cultures of *Microcystis aeruginosa* PCC 7806. Front. Microbiol..

[383] Rodell M., Famiglietti J.S., Wiese D.N., Reager J.T., Beaudoing H.K., Landerer F.W., Lo M.H. (2018). Emerging trends in global freshwater availability. Nature.

[384] Torremorell A., Hegoburu C., Brandimarte A.L., Rodrigues E.H.C., Pompêo M., da Silva S.C., Moschini-Carlos V., Caputo L., Fierro P., Mojica J.I. (2021). Current and future threats for ecological quality management of South American freshwater ecosystems. Inland Waters.

[385] Mignot A., Ferrari R., Claustre H. (2018). Floats with bio-optical sensors reveal what processes trigger the North Atlantic bloom. Nat. Commun..

[386] Cai P., Cai Q., He F., Huang Y., Tian C., Wu X., Wang C., Xiao B. (2021). Flexibility of Microcystisoverwintering strategy in response to winter temperatures. Microorganisms.

[387] Gobler C.J. (2020). Climate change and harmful algal blooms: Insights and perspective. Harmful Algae.

[388] Griffith A.W., Gobler C.J. (2020). Harmful algal blooms: A climate change co-stressor in marine and freshwater ecosystems. Harmful Algae.

[389] Lad A., Breidenbach J.D., Su R.C., Murray J., Kuang R., Mascarenhas A., Najjar J., Patel S., Hegde P., Youssef M. (2022). As we drink and breathe: Adverse health effects of microcystins and other harmful algal bloom toxins in the liver, gut, lungs and beyond. Life.

[390] Chia M.A., Kramer B.J., Jankowiak J.G., Bittencourt-Oliveira M.D.C., Gobler C.J. (2019). The Individual and Combined Effects of the Cyanotoxins, Anatoxin-a and Microcystin-LR, on the Growth, Toxin Production, and Nitrogen Fixation of Prokaryotic and Eukaryotic Algae. Toxins.

[391] Zepernick B.N., Denison E.R., Chaffin J.D., Bullerjahn G.S., Pennacchio C.P., Frenken T., Peck D.H., Anderson J.T., Niles D., Zastepa A. (2022). Metatranscriptomic sequencing of winter and spring planktonic communities from Lake Erie, a Laurentian Great Lake. Microbiol. Resour. Announc..

[392] Howard M.D., Smith J., Caron D.A., Kudela R.M., Loftin K., Hayashi K., Fadness R., Fricke S., Kann J., Roethler M. (2022). Integrative monitoring strategy for marine and freshwater harmful algal blooms and toxins across the freshwater to marine continuum. Integr. Environ. Assess. Manag..

[393] Gaget V., Lau M., Sendall B., Froscio S., Humpage A.R. (2017). Cyanotoxins: Which detection technique for an optimus risk assessment?. Water Res..

